# Supplementary material for: Characterisation of Canine and Feline Breast Tumours, Their Metastases, and Corresponding Primary Cell Lines Using LA-REIMS and DESI-MS Imaging
Source: Int J Mol Sci. 2024 Jul 15;25(14):7752. doi: 10.3390/ijms25147752 (PMC11277125; doi:10.3390/ijms25147752)
Supplement: Supplementary file 1 [file ijms-25-07752-s001.zip › ijms-2943399-supplementary.pdf]

SUPPLEMENTARY INFORMATION

Figure S1. LA-REIMS imaging

Analysis of the tissue samples with LA-REIMS imaging. (A) Normal (N), tumorous (T) and necrotic (Ne) tissue parts were identified by pathological annotation. (B) kNN algorithm was used to identify clusters and the associated peak lists. (C) Images were generated based on the peak lists, selecting the *m/z* ions shown in the figures. (D) Confusion matrices containing true positive (TP), true negative (TN), false positive (FP) and false negative (FN) classifications were obtained. Overlap between the pathological annotation and the imaging was determined by sensitivity and accuracy.

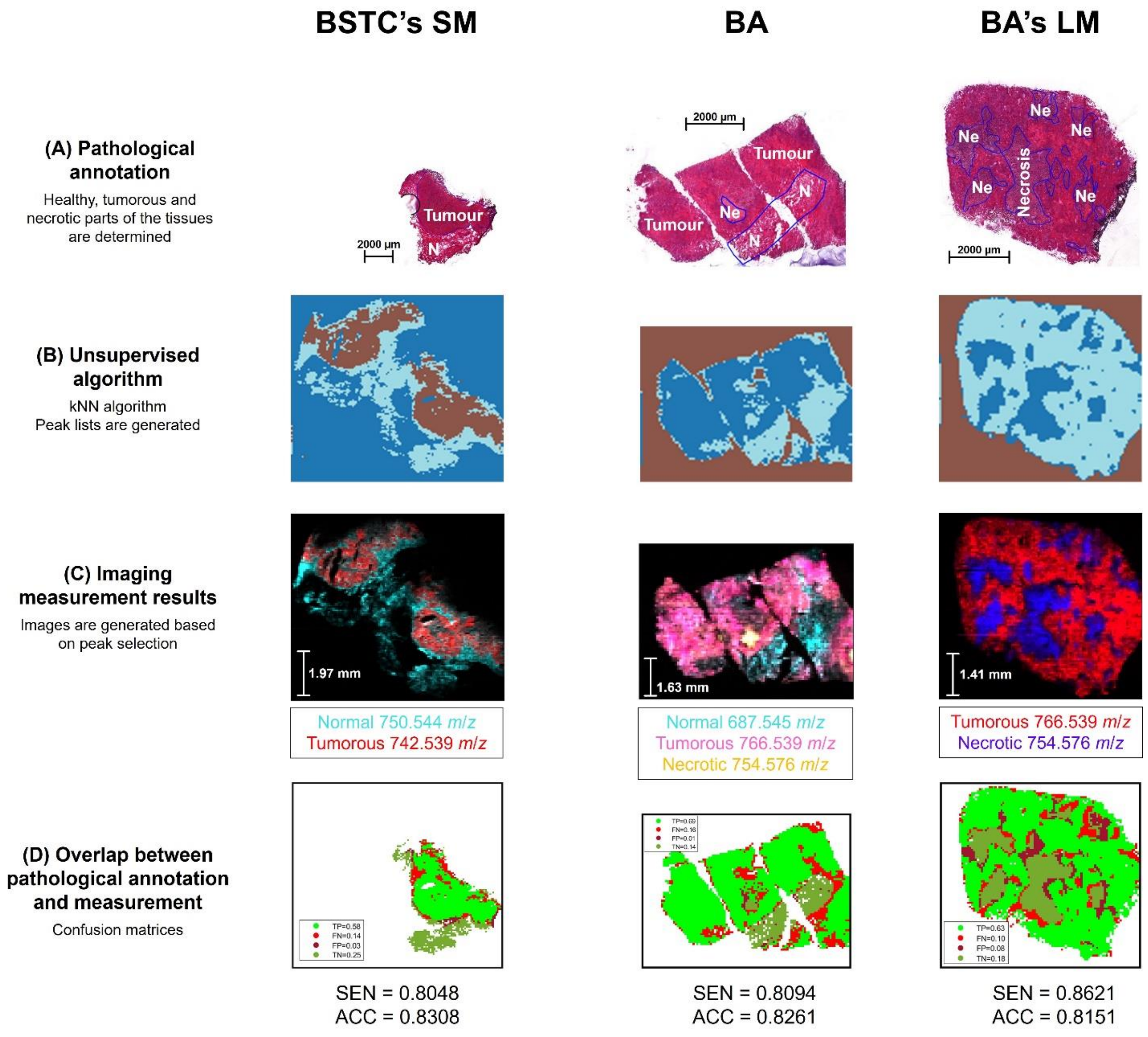

Figure S2. DESI-MSI

Analysis of the tissue samples with DESI-MSI. (A) Normal (N), tumorous (T) and necrotic (Ne) tissue parts were identified by pathological annotation. (B) kNN algorithm was used to identify clusters and the associated peak lists. (C) Images were generated based on the peak lists, selecting the *m*/*z* ions shown in the figures. (D) Confusion matrices containing true positive (TP), true negative (TN), false positive (FP) and false negative (FN) classifications were obtained. Overlap between the pathological annotation and the imaging was determined by sensitivity and accuracy.

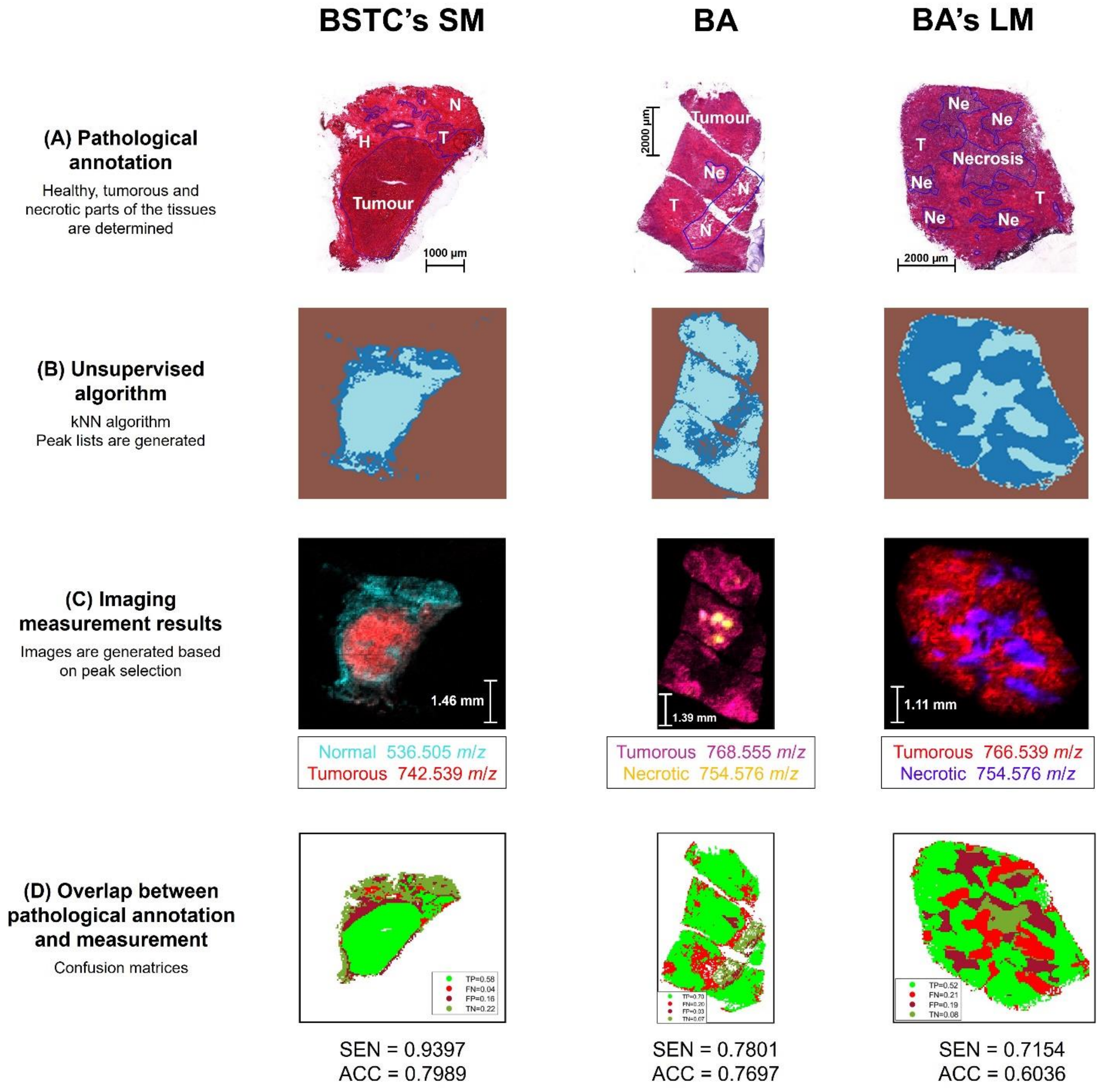

Figure S3. A-D

Classification results of the primary and metastatic cancer tissues for LA-REIMS imaging (A) when all spectra of the two metastases were left out from the training set, and (D) when the two primary tumours were left out, and for DESI-MSI (B) when all spectra of the two metastases were left out, and (C) when the two primary tumours were left out.

A)

LA-REIMS	BA tumour tissue	BA's LM tumour tissue	BSTC tumour tissue	BSTC's SM tumour tissue	Outlier	Total	Correct identification
BA tumour tissue	0	0	0	0	0	0	–
BA's LM tumour tissue	200	0	0	0	0	200	100%
BSTC tumour tissue	0	0	0	0	0	0	–
BSTC's SM tumour tissue	0	0	238	0	0	238	100%
Total	200	0	238	0	0	438	100%

B)

DESI	BA tumour tissue	BA's LM tumour tissue	BSTC tumour tissue	BSTC's SM tumour tissue	Outlier	Total	Correct identification
BA tumour tissue	0	0	0	0	0	0	–
BA's LM tumour tissue	224	0	0	0	0	224	100%
BSTC tumour tissue	0	0	0	0	0	0	–
BSTC's SM tumour tissue	0	0	210	0	0	210	100%
Total	224	0	210	0	0	434	100%

C)

DESI	BA tumour tissue	BA's LM tumour tissue	BSTC tumour tissue	BSTC's SM tumour tissue	Outlier	Total	Correct identification
BA tumour tissue	0	202	0	0	0	202	100%
BA's LM tumour tissue	0	0	0	0	0	0	–
BSTC tumour tissue	0	0	0	217	0	217	100%
BSTC's SM tumour tissue	0	0	0	0	0	0	–
Total	0	202	0	217	0	419	100%

D)

LA-REIMS	BA tumour tissue	BA's LM tumour tissue	BSTC tumour tissue	BSTC's SM tumour tissue	Outlier	Total	Correct identification
BA tumour tissue	0	166	0	0	0	166	100%
BA's LM tumour tissue	0	0	0	0	0	0	–
BSTC tumour tissue	2	29	0	163	0	195	83.59%
BSTC's SM tumour tissue	0	0	0	0	0	0	–
Total	2	195	0	163	0	361	91.14%

Figure S4. A and B

Mass spectra (average of 20 scans, 600-900 *m*/*z* range) of the immortalised cell samples by (A) LA-REIMS imaging and (B) DESI-MSI.

A) LA-REIMS imaging

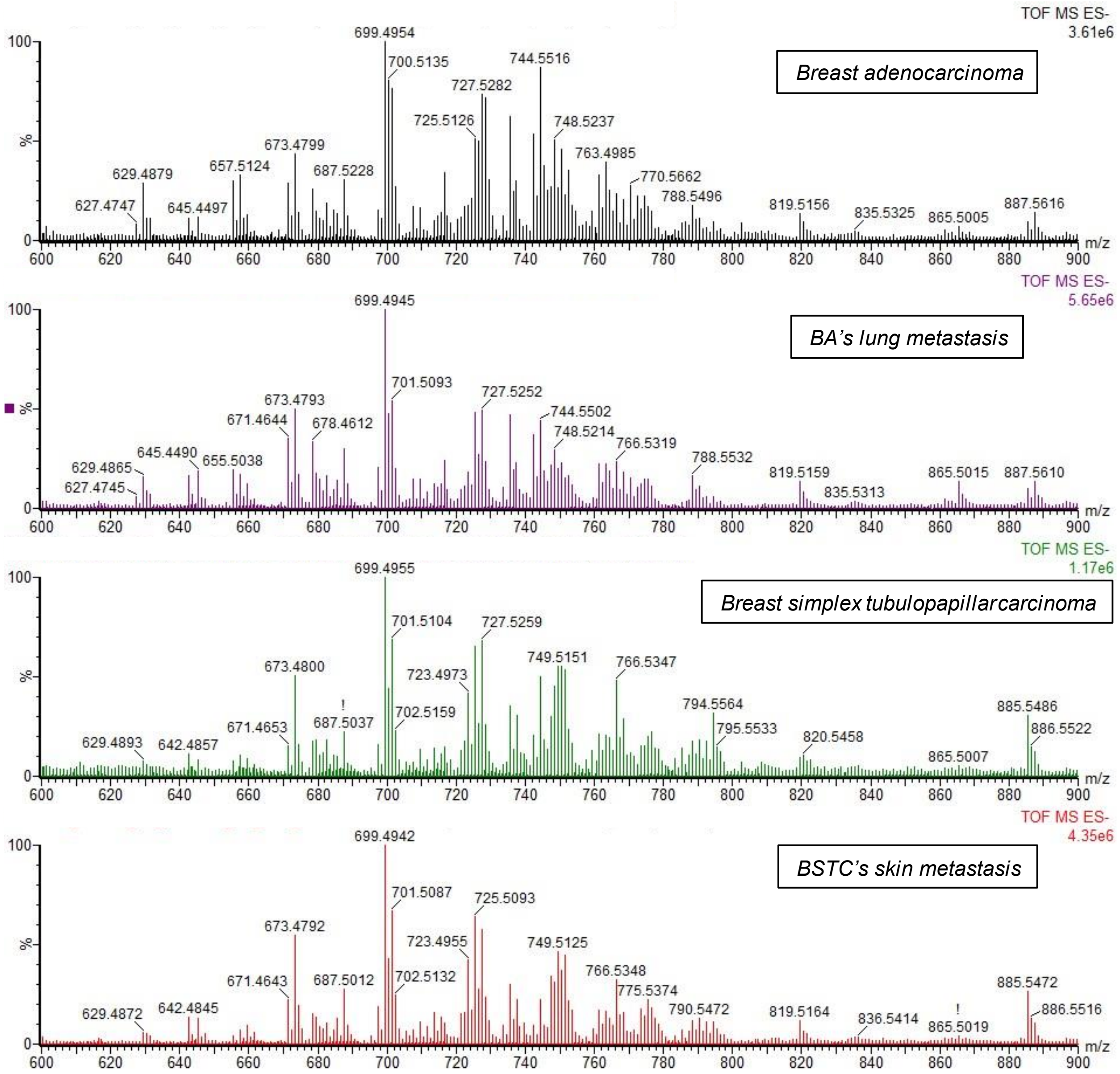

B) DESI-MSI

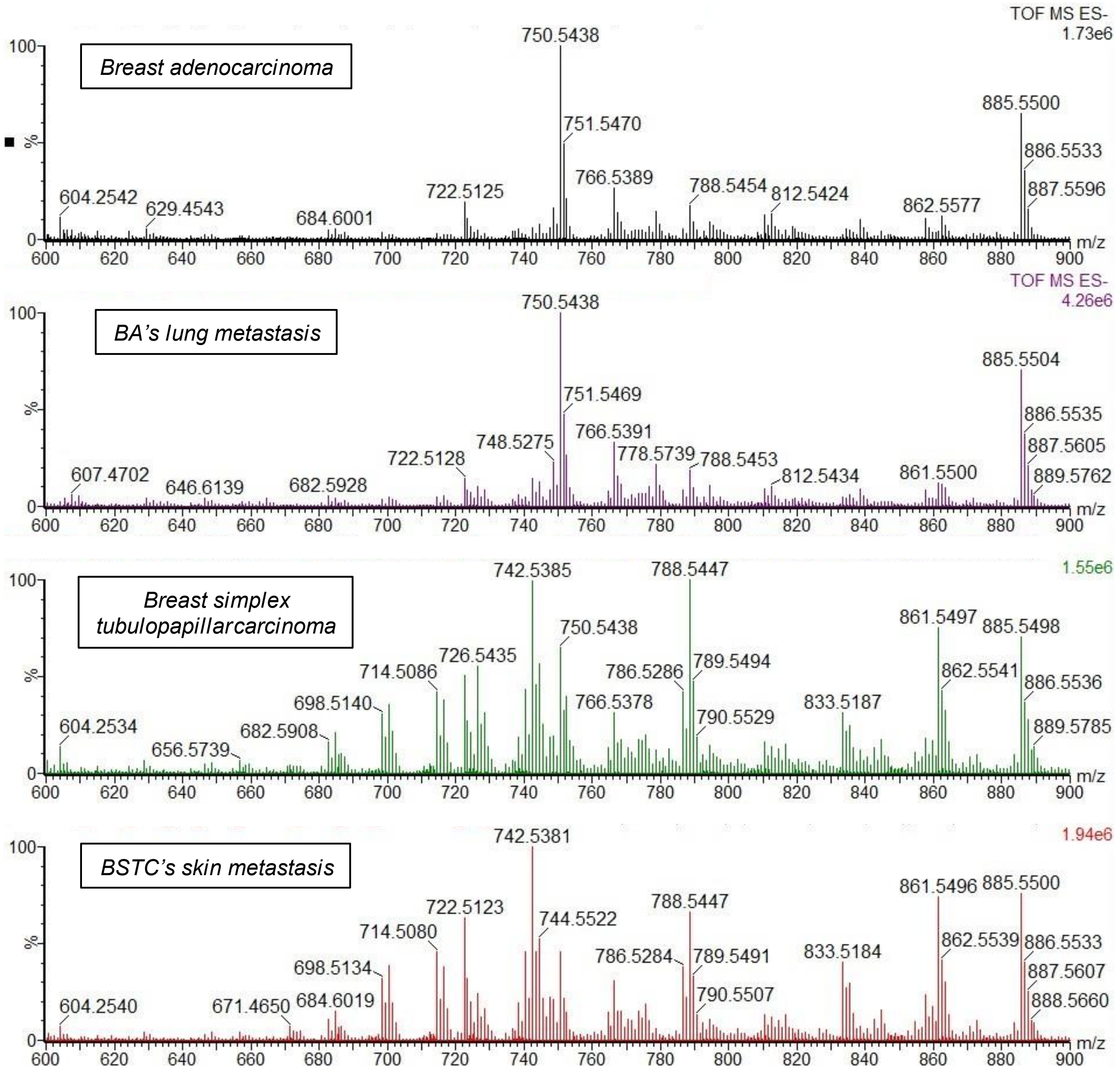
Breast adenocarcinoma
TOF MS ES-
1.73e6
750.5438
751.5470
766.5389
788.5454
812.5424
885.5500
886.5533
887.5596
862.5577
722.5125
604.2542
629.4543
684.6001
BA's lung metastasis
TOF MS ES-
4.26e6
750.5438
751.5469
766.5391
778.5739
788.5453
748.5275
722.5128
812.5434
861.5500
885.5504
886.5535
887.5605
889.5762
607.4702
646.6139
682.5928
Breast simplex tubulopapillarcarcinoma
1.55e6
742.5385
788.5447
750.5438
726.5435
714.5086
698.5140
786.5286
789.5494
766.5378
790.5529
833.5187
861.5497
885.5498
862.5541
886.5536
889.5785
604.2534
656.5739
682.5908
BSTC's skin metastasis
1.94e6
742.5381
722.5123
744.5522
714.5080
698.5134
788.5447
786.5284
789.5491
790.5507
833.5184
861.5496
885.5500
862.5539
886.5533
887.5607
888.5660
604.2540
671.4650
684.6019
%
m/z

Figure S5.

Mass spectra of the breast simplex tubulopapillar carcinoma cell line and its parental tumour tissue (average of 30 scans) measured by DESI-MSI. There is a rich and distinct lipidomic signal in the 600-900 *m*/*z* region, while the metabolic fingerprint of smaller molecules, including fatty acids, is significantly different in the 250-350 *m*/*z* region. Mass spectra were lock mass corrected to the internal standard leucine enkephalin (*m*/*z* 554.2615), which is marked with an asterisk.

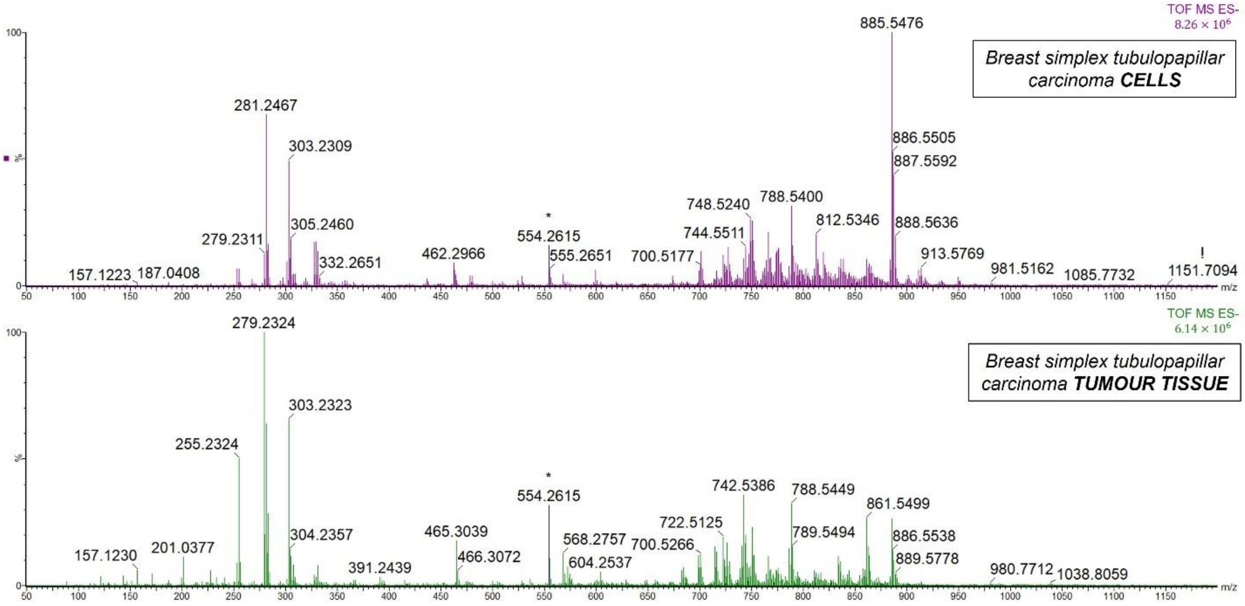

Figure S6. A-D

The most abundant lipids identified in (A) BA tumour, (B) LM tumour, (C) BSTC tumour, (D) SM tumour and their corresponding cell lines in the case of LA-REIMS imaging, highlighting the lipids identified in both.

A)

Most abundant lipids identified for BA tumour

m/z (accurate)	Lipids	Adduct
673.481	PA(34:1)	$[M-H]^-$
678.472	HexCer (30:1;O2)	$[M+Cl]^-$
687.545	CerPE(36:1;O2)	$[M-H]^-$
699.497	PA(36:2)	$[M-H]^-$
722.513	PE(O-36:5)<br>PE(P-36:4)	$[M-H]^-$<br>$[M-H]^-$
744.555	PE(36:1)	$[M-H]^-$
748.529	PE(O-38:6)<br>PE(P-38:5)	$[M-H]^-$<br>$[M-H]^-$
750.544	PE(O-38:5)<br>PE(P-38:4)	$[M-H]^-$<br>$[M-H]^-$
752.560	PE(O-38:4)<br>PE(P-38:3)	$[M-H]^-$<br>$[M-H]^-$
766.539	PE(38:4)	$[M-H]^-$
768.555	PE(38:3)	$[M-H]^-$
778.576	PE(O-40:5)<br>PE(P-40:4)	$[M-H]^-$<br>$[M-H]^-$
788.545	PS(36:1)	$[M-H]^-$
794.571	PE(40:4)<br>PC(38:4)	$[M-H]^-$<br>$[M-CH_3]^-$
885.550	PI(38:4)	$[M-H]^-$

Most abundant lipids identified for BA cell line

m/z (accurate)	Lipids	Adduct
673.481	PA(34:1)	$[M-H]^-$
699.497	PA(36:2)	$[M-H]^-$
701.513	PA(36:1)	$[M-H]^-$
716.524	PE(34:1)	$[M-H]^-$
725.513	PA(38:3)	$[M-H]^-$
727.528	PA(38:2)	$[M-H]^-$
729.544	PA(38:1)	$[M-H]^-$
735.474	PA(36:2)<br>PE(34:1)	$[M+Cl]^-$<br>$[M-NH_3+Cl]^-$
742.539	PE(36:2)	$[M-H]^-$
744.555	PE(36:1)	$[M-H]^-$
748.529	PE(O-38:6)<br>PE(P-38:5)	$[M-H]^-$<br>$[M-H]^-$
750.544	PE(O-38:5)<br>PE(P-38:4)	$[M-H]^-$<br>$[M-H]^-$
752.560	PE(O-38:4)<br>PE(P-38:3)	$[M-H]^-$<br>$[M-H]^-$
761.489	PA(38:3)<br>PS(O-38:6)<br>PS(P-38:5)	$[M-H]^-$<br>$[M-NH_3-CH_3]^-$<br>$[M-NH_3-CH_3]^-$
763.505	PA(38:2)<br>PS(O-38:5)<br>PS(P-38:4)	$[M+Cl]^-$<br>$[M-NH_3-CH_3]^-$<br>$[M-NH_3-CH_3]^-$

B)

Most abundant lipids identified for LM tumour

m/z (accurate)	Lipids	Adduct
678.472	HexCer (30:1;O2)	$[M+Cl]^-$
697.481	PA(36:3)	$[M-H]^-$
699.497	PA(36:2)	$[M-H]^-$
722.513	PE(O-36:5)	$[M-H]^-$
	PE(P-36:4)	$[M-H]^-$
735.474	PA(36:2)	$[M+Cl]^-$
	PE(34:1)	$[M-NH_3+Cl]^-$
742.539	PE(36:2)	$[M-H]^-$
744.555	PE(36:1)	$[M-H]^-$
748.529	PE(O-38:6)	$[M-H]^-$
	PE(P-38:5)	$[M-H]^-$
750.544	PE(O-38:5)	$[M-H]^-$
	PE(P-38:4)	$[M-H]^-$
752.560	PE(O-38:4)	$[M-H]^-$
	PE(P-38:3)	$[M-H]^-$
766.539	PE(38:4)	$[M-H]^-$
768.555	PE(38:3)	$[M-H]^-$
778.576	PE(O-40:5)	$[M-H]^-$
	PE(P-40:4)	$[M-H]^-$
794.571	PE(40:4)	$[M-H]^-$
	PC(38:4)	$[M-CH_3]^-$
885.550	PI(38:4)	$[M-H]^-$

Most abundant lipids identified for LM cell line

m/z (accurate)	Lipids	Adduct
671.466	PA(34:2)	$[M-H]^-$
673.481	PA(34:1)	$[M-H]^-$
699.497	PA(36:2)	$[M-H]^-$
701.513	PA(36:1)	$[M-H]^-$
716.524	PE(34:1)	$[M-H]^-$
725.513	PA(38:3)	$[M-H]^-$
727.528	PA(38:2)	$[M-H]^-$
735.474	PA(36:2)	$[M+Cl]^-$
	PE(34:1)	$[M-NH_3+Cl]^-$
737.489	PA(36:1)	$[M+Cl]^-$
742.539	PE(36:2)	$[M-H]^-$
744.555	PE(36:1)	$[M-H]^-$
748.529	PE(O-38:6)	$[M-H]^-$
	PE(P-38:5)	$[M-H]^-$
750.543	PE(O-38:5)	$[M-H]^-$
	PE(P-38:4)	$[M-H]^-$
761.489	PA(38:3)	$[M+Cl]^-$
	PS(O-38:6)	$[M-NH_3-CH_3]^-$
	PS(P-38:5)	$[M-NH_3-CH_3]^-$
766.539	PE(38:4)	$[M-H]^-$

C)

Most abundant lipids identified for BSTC tumour

m/z (accurate)	Lipids	Adduct
678.472	HexCer (30:1;O2)	$[M+Cl]^-$
697.481	PA(36:3)	$[M-H]^-$
699.497	PA(36:2)	$[M-H]^-$
701.513	PA(36:1)	$[M-H]^-$
714.508	PE(34:2)	$[M-H]^-$
716.524	PE(34:1)	$[M-H]^-$
722.513	PE(O-36:5)	$[M-H]^-$
	PE(P-36:4)	$[M-H]^-$
725.513	PA(38:3)	$[M-H]^-$
733.458	PA(36:3)	$[M+Cl]^-$
	PE(34:2)	$[M-NH_3+Cl]^-$
735.473	PA(36:2)	$[M+Cl]^-$
	PE(34:1)	$[M-NH_3+Cl]^-$
740.524	PE(36:3)	$[M-H]^-$
742.539	PE(36:2)	$[M-H]^-$
744.555	PE(36:1)	$[M-H]^-$
750.544	PE(O-38:5)	$[M-H]^-$
	PE(P-38:4)	$[M-H]^-$
766.539	PE(38:4)	$[M-H]^-$

Most abundant lipids identified for BSTC cell line

m/z (accurate)	Lipids	Adduct
673.481	PA(34:1)	$[M-H]^-$
699.497	PA(36:2)	$[M-H]^-$
701.513	PA(36:1)	$[M-H]^-$
723.497	PA(38:4)	$[M-H]^-$
725.513	PA(38:3)	$[M-H]^-$
727.528	PA(38:2)	$[M-H]^-$
735.474	PA(36:2)	$[M+Cl]^-$
	PE(34:1)	$[M-NH_3+Cl]^-$
744.555	PE(36:1)	$[M-H]^-$
747.497	PA(40:6)	$[M-H]^-$
	PE(38:5)	$[M-NH_4^+]^-$
748.529	PE(O-38:6)	$[M-H]^-$
	PE(P-38:5)	$[M-H]^-$
749.513	PA(40:5)	$[M-H]^-$
	PE(38:4)	$[M-NH_4^+]^-$
750.544	PE(O-38:5)	$[M-H]^-$
	PE(P-38:4)	$[M-H]^-$
751.528	PA(40:4)	$[M-H]^-$
	PE(38:3)	$[M-NH_4^+]^-$
766.539	PE(38:4)	$[M-H]^-$
885.550	PI(38:4)	$[M-H]^-$

D)

Most abundant lipids identified for SM tumour

m/z (accurate)	Lipids	Adduct
684.607	Cer(42:1;O2)	$[M+Cl]^-$
698.513	PE(P-34:2)	$[M-H]^-$
699.497	PA(36:2)	$[M-H]^-$
700.529	PE(O-34:2)	$[M-H]^-$
	PE(P-34:1)	$[M-H]^-$
714.508	PE(34:2)	$[M-H]^-$
716.524	PE(34:1)	$[M-H]^-$
722.513	PE(O-36:5)	$[M-H]^-$
	PE(P-36:4)	$[M-H]^-$
726.544	PE(O-36:3)	$[M-H]^-$
	PE(P-36:2)	$[M-H]^-$
733.458	PA(36:3)	$[M+Cl]^-$
	PE(34:2)	$[M-NH_3+Cl]^-$
735.473	PA(36:2)	$[M+Cl]^-$
	PE(34:1)	$[M-NH_3+Cl]^-$
740.524	PE(36:3)	$[M-H]^-$
742.539	PE(36:2)	$[M-H]^-$
744.555	PE(36:1)	$[M-H]^-$
750.544	PE(O-38:5)	$[M-H]^-$
	PE(P-38:4)	$[M-H]^-$
766.539	PE(38:4)	$[M-H]^-$

Most abundant lipids identified for SM cell line

m/z (accurate)	Lipids	Adduct
673.481	PA(34:1)	$[M-H]^-$
699.497	PA(36:2)	$[M-H]^-$
701.513	PA(36:1)	$[M-H]^-$
723.497	PA(38:4)	$[M-H]^-$
725.513	PA(38:3)	$[M-H]^-$
727.528	PA(38:2)	$[M-H]^-$
735.474	PA(36:2)	$[M+Cl]^-$
	PE(34:1)	$[M-NH_3+Cl]^-$
744.555	PE(36:1)	$[M-H]^-$
747.497	PA(40:6)	$[M-H]^-$
	PE(38:5)	$[M-NH_4^+]^-$
748.529	PE(O-38:6)	$[M-H]^-$
	PE(P-38:5)	$[M-H]^-$
749.513	PA(40:5)	$[M-H]^-$
	PE(38:4)	$[M-NH_4^+]^-$
750.544	PE(O-38:5)	$[M-H]^-$
	PE(P-38:4)	$[M-H]^-$
751.528	PA(40:4)	$[M-H]^-$
	PE(38:3)	$[M-NH_4^+]^-$
766.539	PE(38:4)	$[M-H]^-$
885.550	PI(38:4)	$[M-H]^-$

Figure S7. A-D

The most abundant lipids identified in (A) BA tumour, (B) LM tumour, (C) BSTC tumour, (D) SM tumour and their corresponding cell lines in the case of DESI-MSI, highlighting the lipids identified in both.

A)

Most abundant lipids identified forBA tumour

m/z (accurate)	Lipids	Adduct
722.513	PE(O-36:5)	[M-H]$^-$
	PE(P-36:4)	[M-H]$^-$
748.529	PE(O-38:6)	[M-H]$^-$
	PE(P-38:5)	[M-H]$^-$
750.544	PE(O-38:5)	[M-H]$^-$
	PE(P-38:4)	[M-H]$^-$
752.560	PE(O-38:4)	[M-H]$^-$
	PE(P-38:3)	[M-H]$^-$
766.539	PE(38:4)	[M-H]$^-$
778.576	PE(O-40:5)	[M-H]$^-$
	PE(P-40:4)	[M-H]$^-$
788.545	PS(36:1)	[M-H]$^-$
794.571	PE(40:4)	[M-H]$^-$
	PC(38:4)	[M-CH_3]$^-$
810.529	PS(38:4)	[M-H]$^-$
812.545	PS(38:3)	[M-H]$^-$
838.560	PS(40:4)	[M-H]$^-$
	PC(36:5)	[M+OAc]$^-$
857.519	PI(36:4)	[M-H]$^-$
862.560	PS(42:6)	[M-H]$^-$
885.550	PI(38:4)	[M-H]$^-$
887.566	PI(38:3)	[M-H]$^-$

Most abundant lipids identified for BA cell line

m/z (accurate)	Lipids	Adduct
701.513	PA(36:1)	[M-H]$^-$
728.560	PE(P-36:1)	[M-H]$^-$
744.555	PE(36:1)	[M-H]$^-$
747.497	PA(40:6)	[M-H]$^-$
	PE(38:5)	[M-NH_4^+]$^-$
748.529	PE(O-38:6)	[M-H]$^-$
	PE(P-38:5)	[M-H]$^-$
750.544	PE(O-38:5)	[M-H]$^-$
	PE(P-38:4)	[M-H]$^-$
774.544	PE(P-40:6)	[M-H]$^-$
788.545	PS(36:1)	[M-H]$^-$
819.518	PG(40:7)	[M-H]$^-$
835.534	PI(34:1)	[M-H]$^-$
861.550	PI(36:2)	[M-H]$^-$
	PG(40:4)	[M+Cl]$^-$
863.566	PI(36:1)	[M-H]$^-$
	PG(40:3)	[M+Cl]$^-$
885.550	PI(38:4)	[M-H]$^-$
887.566	PI(38:3)	[M-H]$^-$
889.581	PI(38:2)	[M-H]$^-$
	PG(42:2)	[M+Cl]$^-$

B)

Most abundant lipids identified for LM tumour

m/z (accurate)	Lipids	Adduct
722.513	PE(O-36:5)	$[M-H]^-$
	PE(P-36:4)	$[M-H]^-$
748.529	PE(O-38:6)	$[M-H]^-$
	PE(P-38:5)	$[M-H]^-$
750.544	PE(O-38:5)	$[M-H]^-$
	PE(P-38:4)	$[M-H]^-$
752.560	PE(O-38:4)	$[M-H]^-$
	PE(P-38:3)	$[M-H]^-$
766.539	PE(38:4)	$[M-H]^-$
768.555	PE(38:3)	$[M-H]^-$
778.576	PE(O-40:5)	$[M-H]^-$
	PE(P-40:4)	$[M-H]^-$
788.545	PS(36:1)	$[M-H]^-$
794.571	PE(40:4)	$[M-H]^-$
	PC(38:4)	$[M-CH_3]^-$
810.529	PS(38:4)	$[M-H]^-$
812.545	PS(38:3)	$[M-H]^-$
838.560	PS(40:4)	$[M-H]^-$
	PC(36:5)	$[M+OAc]^-$
862.560	PS(42:6)	$[M-H]^-$
885.550	PI(38:4)	$[M-H]^-$
887.566	PI(38:3)	$[M-H]^-$

Most abundant lipids identified for LM cell line

m/z (accurate)	Lipids	Adduct
701.513	PA(36:1)	$[M-H]^-$
742.539	PE(36:2)	$[M-H]^-$
744.555	PE(36:1)	$[M-H]^-$
747.497	PA(40:6)	$[M-H]^-$
	PE(38:5)	$[M-NH_4^+]^-$
748.529	PE(O-38:6)	$[M-H]^-$
	PE(P-38:5)	$[M-H]^-$
750.544	PE(O-38:5)	$[M-H]^-$
	PE(P-38:4)	$[M-H]^-$
766.539	PE(38:4)	$[M-H]^-$
774.544	PE(P-40:6)	$[M-H]^-$
788.545	PS(36:1)	$[M-H]^-$
812.545	PS(38:3)	$[M-H]^-$
819.518	PG(40:7)	$[M-H]^-$
861.550	PI(36:2)	$[M-H]^-$
	PG(40:4)	$[M+Cl]^-$
885.550	PI(38:4)	$[M-H]^-$
887.566	PI(38:3)	$[M-H]^-$
889.581	PI(38:2)	$[M-H]^-$
	PG(42:2)	$[M+Cl]^-$

C)

Most abundant lipids identified for BSTC tumour

m/z (accurate)	Lipids	Adduct
700.529	PE(O-34:2)	$[M-H]^-$
	PE(P-34:1)	$[M-H]^-$
714.508	PE(34:2)	$[M-H]^-$
722.513	PE(O-36:5)	$[M-H]^-$
	PE(P-36:4)	$[M-H]^-$
726.544	PE(O-36:3)	$[M-H]^-$
	PE(P-36:2)	$[M-H]^-$
728.560	PE(P-36:1)	$[M-H]^-$
740.524	PE(36:3)	$[M-H]^-$
742.539	PE(36:2)	$[M-H]^-$
744.555	PE(36:1)	$[M-H]^-$
750.544	PE(O-38:5)	$[M-H]^-$
	PE(P-38:4)	$[M-H]^-$
752.560	PE(O-38:4)	$[M-H]^-$
	PE(P-38:3)	$[M-H]^-$
766.539	PE(38:4)	$[M-H]^-$
786.529	PS(36:2)	$[M-H]^-$
788.545	PS(36:1)	$[M-H]^-$
861.550	PI(36:2)	$[M-H]^-$
	PG(40:4)	$[M+Cl]^-$
885.550	PI(38:4)	$[M-H]^-$

Most abundant lipids identified for BSTC cell line

m/z (accurate)	Lipids	Adduct
727.528	PA(38:2)	$[M-H]^-$
744.555	PE(36:1)	$[M-H]^-$
748.529	PE(O-38:6)	$[M-H]^-$
	PE(P-38:5)	$[M-H]^-$
749.513	PA(40:5)	$[M-H]^-$
	PE(38:4)	$[M-NH_4^+]^-$
750.544	PE(O-38:5)	$[M-H]^-$
	PE(P-38:4)	$[M-H]^-$
751.528	PA(40:4)	$[M-H]^-$
	PE(38:3)	$[M-NH_4^+]^-$
766.539	PE(38:4)	$[M-H]^-$
773.534	PG(36:2)	$[M-H]^-$
774.544	PE(P-40:6)	$[M-H]^-$
775.541	PG(36:1)	$[M-H]^-$
776.560	PE(P-40:5)	$[M-H]^-$
788.545	PS(36:1)	$[M-H]^-$
812.545	PS(38:3)	$[M-H]^-$
885.550	PI(38:4)	$[M-H]^-$
887.566	PI(38:3)	$[M-H]^-$

D)

Most abundant lipids identified for SM tumour

m/z (accurate)	Lipids	Adduct
698.513	PE(P-34:2)	$[M-H]^-$
700.529	PE(O-34:2)	$[M-H]^-$
	PE(P-34:1)	$[M-H]^-$
714.508	PE(34:2)	$[M-H]^-$
716.524	PE(34:1)	$[M-H]^-$
722.513	PE(O-36:5)	$[M-H]^-$
	PE(P-36:4)	$[M-H]^-$
740.524	PE(36:3)	$[M-H]^-$
742.539	PE(36:2)	$[M-H]^-$
744.555	PE(36:1)	$[M-H]^-$
750.544	PE(O-38:5)	$[M-H]^-$
	PE(P-38:4)	$[M-H]^-$
766.539	PE(38:4)	$[M-H]^-$
786.529	PS(36:2)	$[M-H]^-$
788.545	PS(36:1)	$[M-H]^-$
833.519	PI(34:2)	$[M-H]^-$
861.550	PI(36:2)	$[M-H]^-$
	PG(40:4)	$[M+Cl]^-$
885.550	PI(38:4)	$[M-H]^-$

Most abundant lipids identified for SM cell line

m/z (accurate)	Lipids	Adduct
744.555	PE(36:1)	$[M-H]^-$
748.529	PE(O-38:6)	$[M-H]^-$
	PE(P-38:5)	$[M-H]^-$
749.526	PA(O-38:2)	$[M+Cl]^-$
	PE(38:4)	$[M-NH_4^+]^-$
750.544	PE(O-38:5)	$[M-H]^-$
	PE(P-38:4)	$[M-H]^-$
751.541	PA(O-38:1)	$[M+Cl]^-$
	PE(38:3)	$[M-NH_4^+]^-$
766.539	PE(38:4)	$[M-H]^-$
773.534	PG(36:2)	$[M-H]^-$
774.544	PE(P-40:6)	$[M-H]^-$
775.541	PG(36:1)	$[M-H]^-$
776.560	PE(P-40:5)	$[M-H]^-$
788.545	PS(36:1)	$[M-H]^-$
812.545	PS(38:3)	$[M-H]^-$
819.518	PG(40:7)	$[M-H]^-$
885.550	PI(38:4)	$[M-H]^-$
887.566	PI(38:3)	$[M-H]^-$

Supplementary Figure S8.

MS/MS spectra of the identified lipids reported in the article.

MS/MS spectrum of *m/z* 671.466 corresponding to PA(34:2).

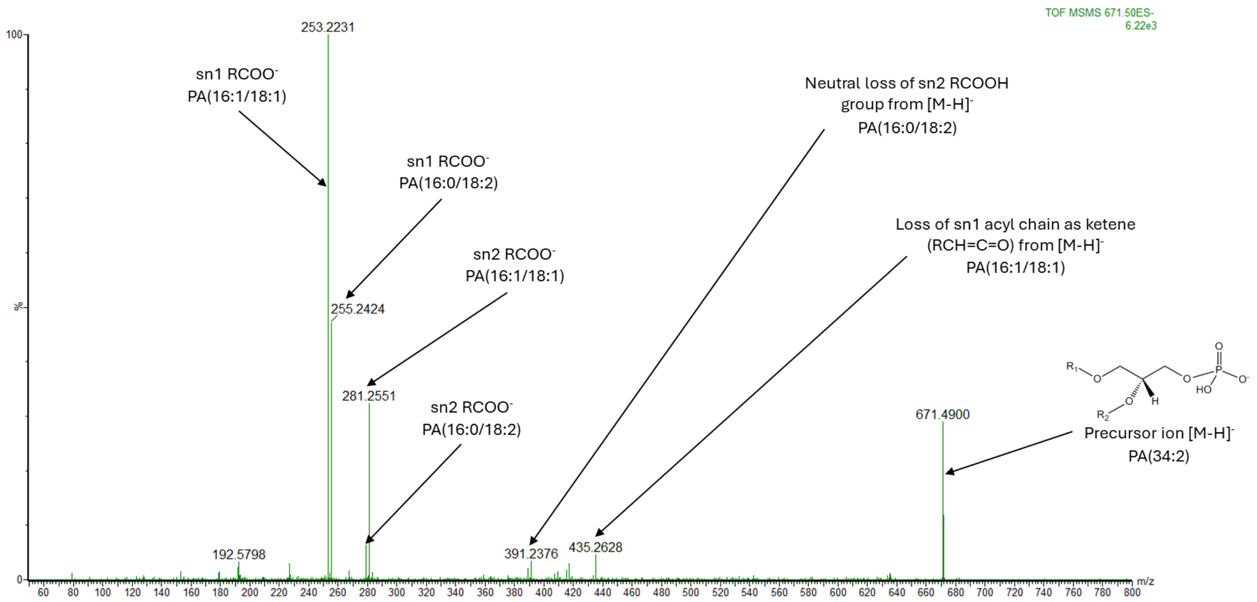

MS/MS spectrum of *m/z* 673.481 corresponding to PA(34:1).

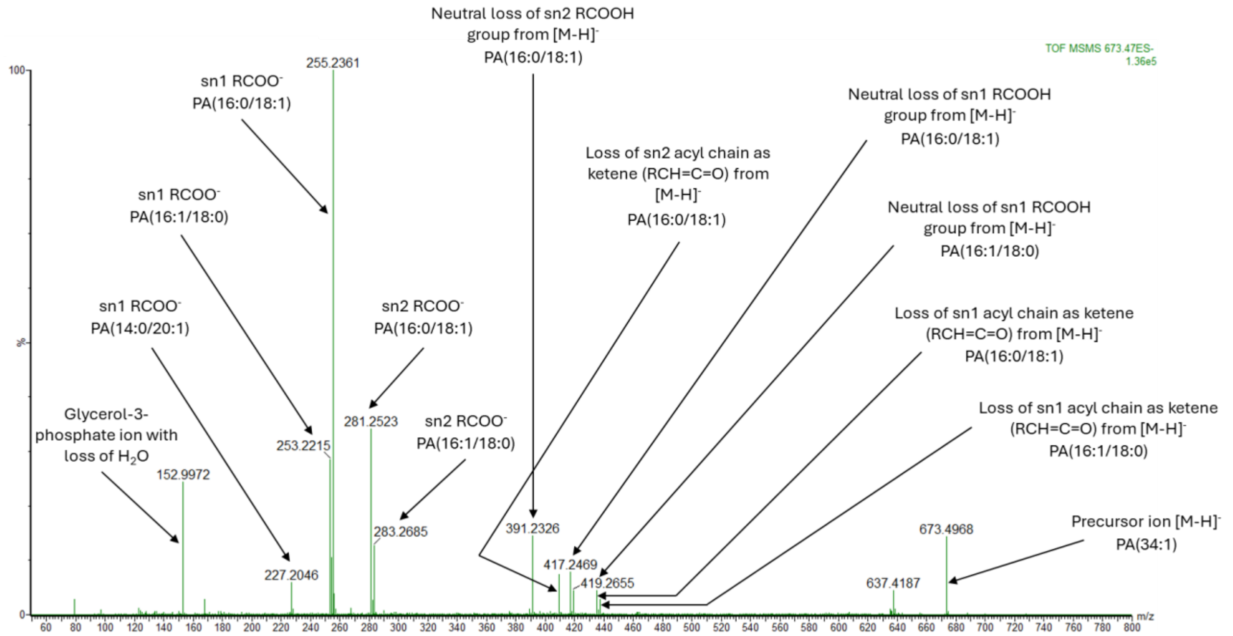

MS/MS spectrum of *m*/*z* 697.481 corresponding to PA(36:3).

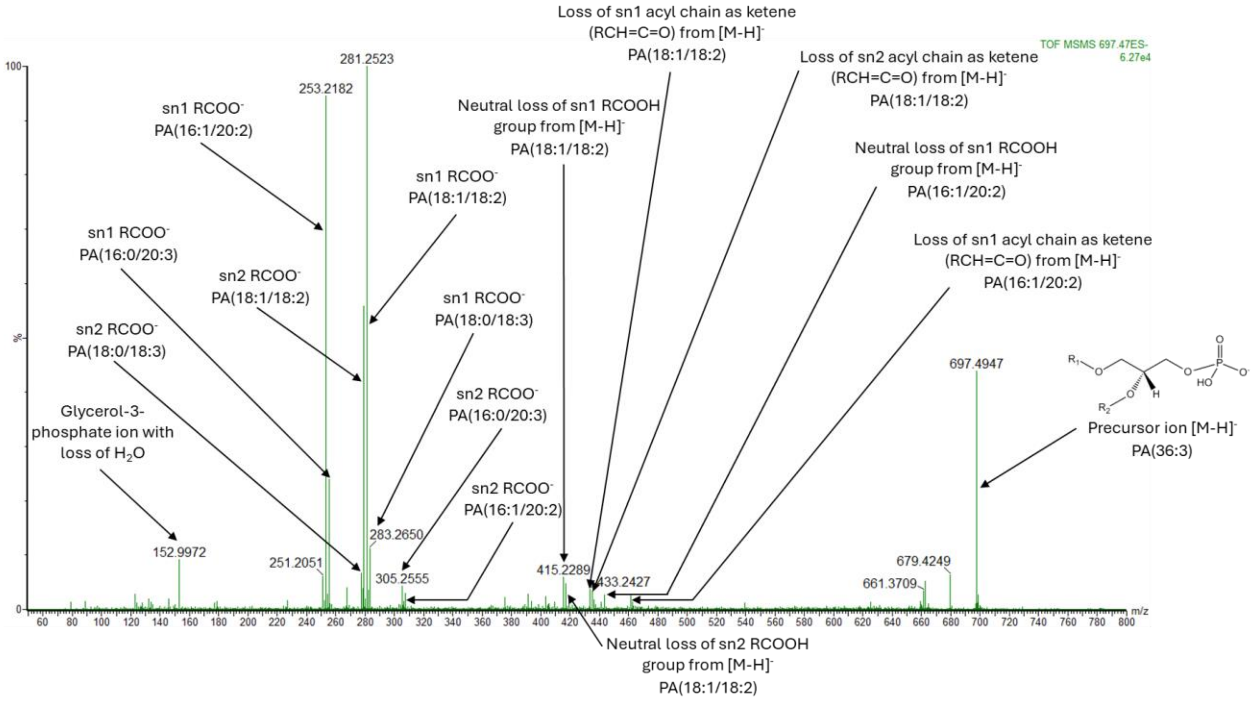

MS/MS spectrum of *m*/*z* 698.513 corresponding to PE(P-34:2).

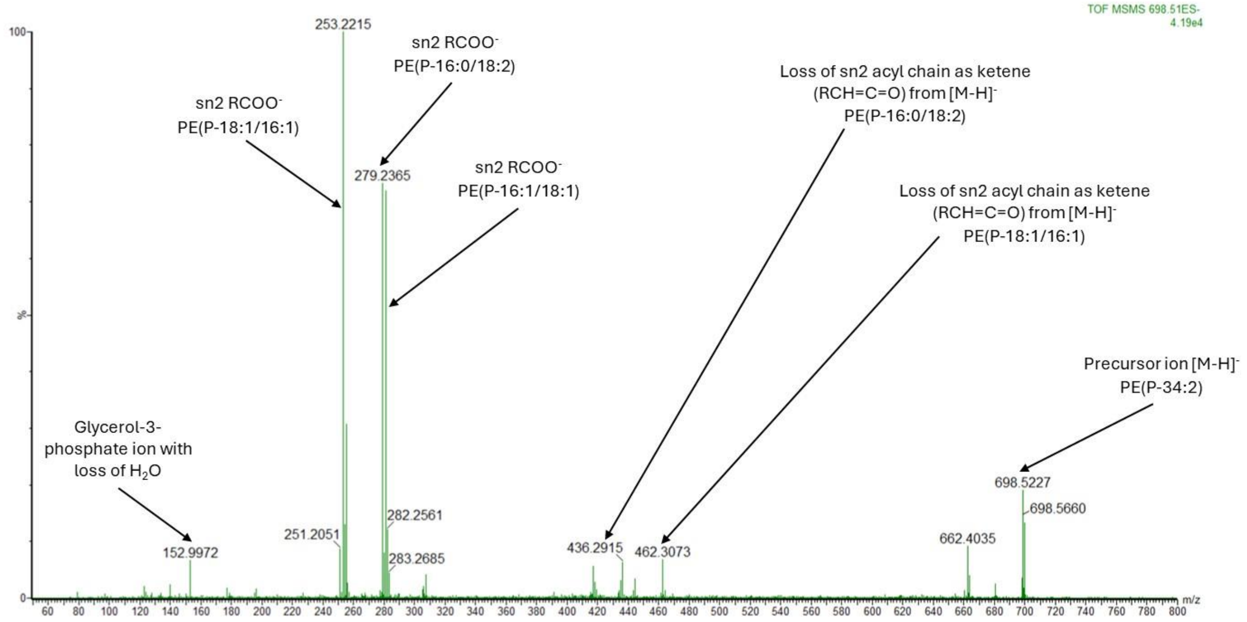

MS/MS spectrum of *m*/*z* 699.497 corresponding to PA(36:2).

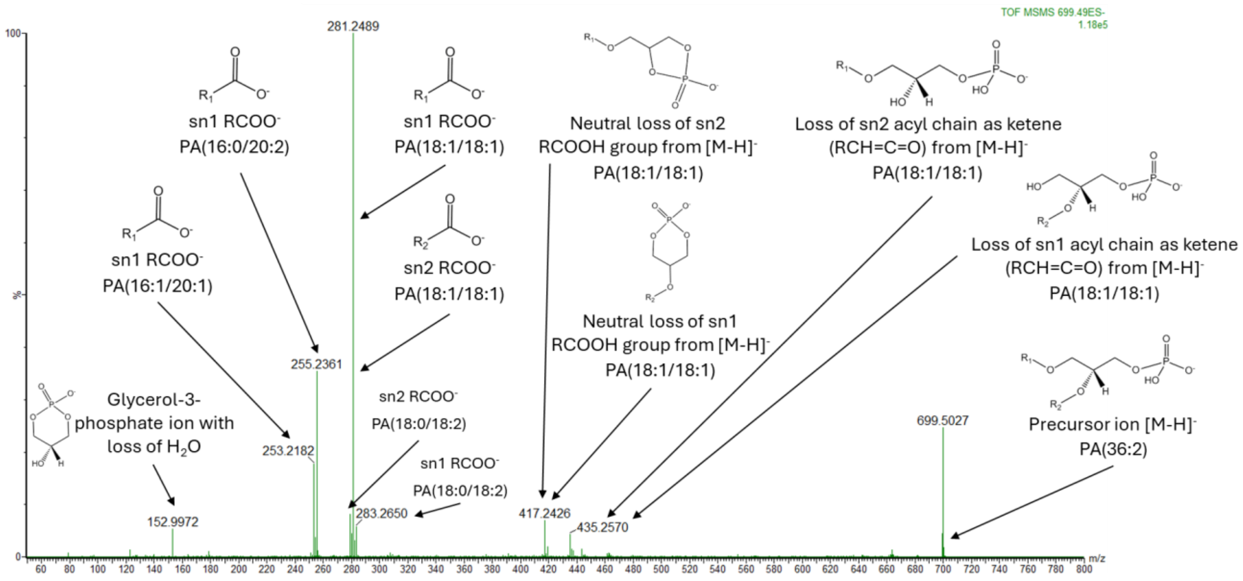

MS/MS spectrum of *m/z* 700.529 corresponding to PE(O-34:2) and PE(P-34:1).

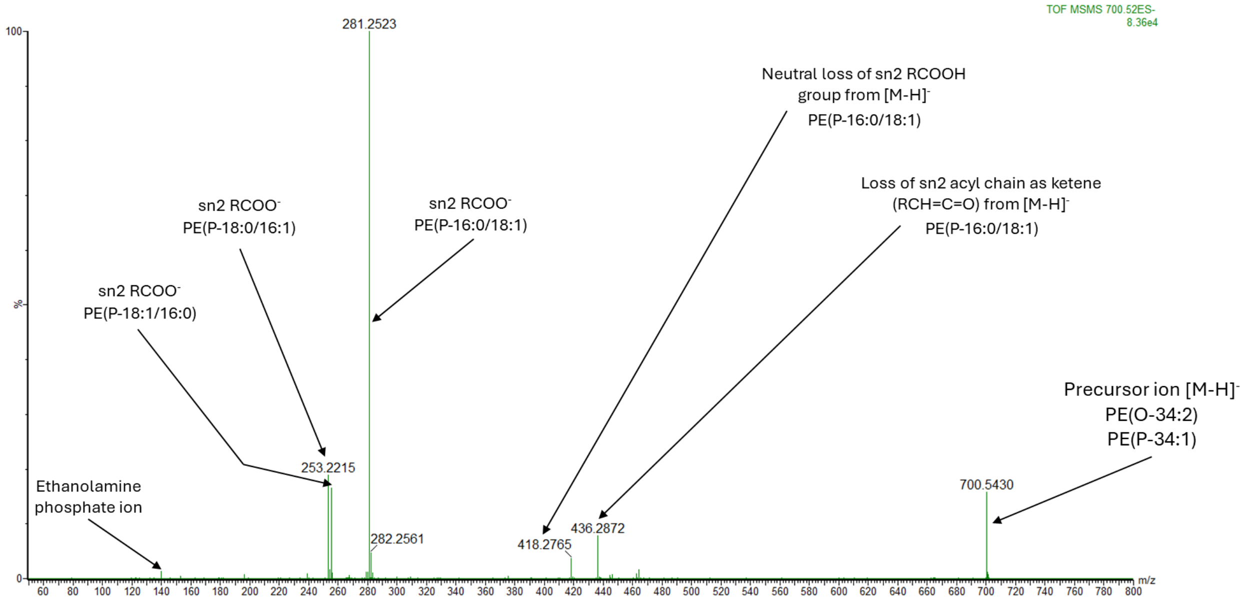

MS/MS spectrum of *m*/*z* 701.513 corresponding to PA(36:1).

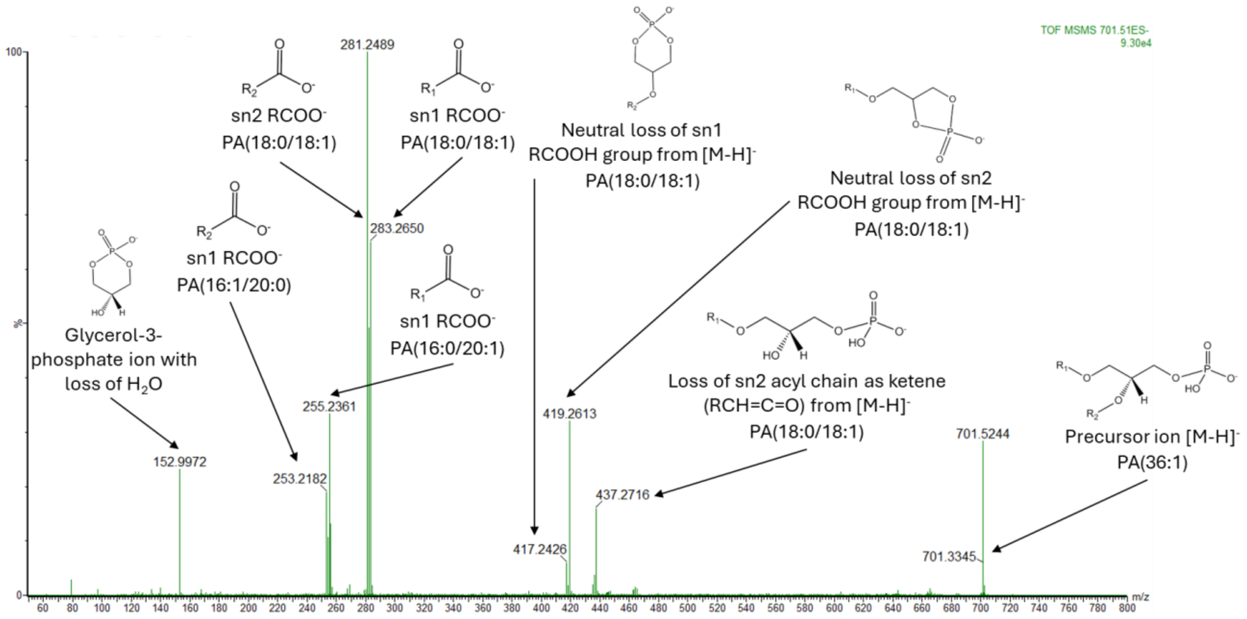

MS/MS spectrum of *m*/*z* 714.508 corresponding to PE(34:2).

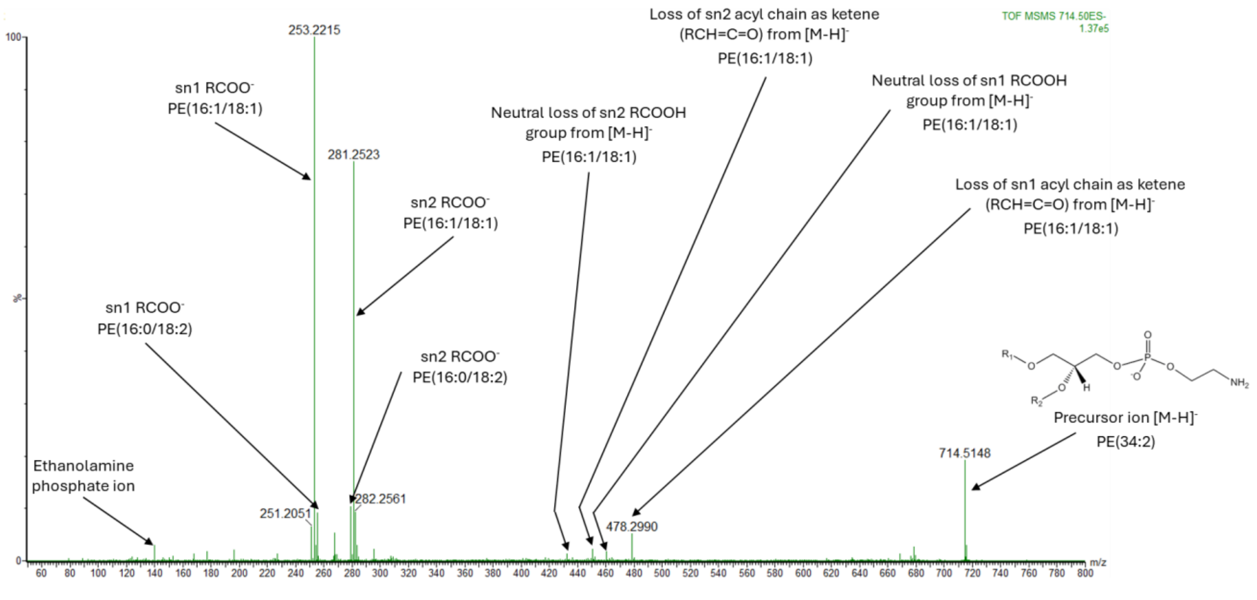

MS/MS spectrum of *m*/*z* 716.524 corresponding to PE(34:1).

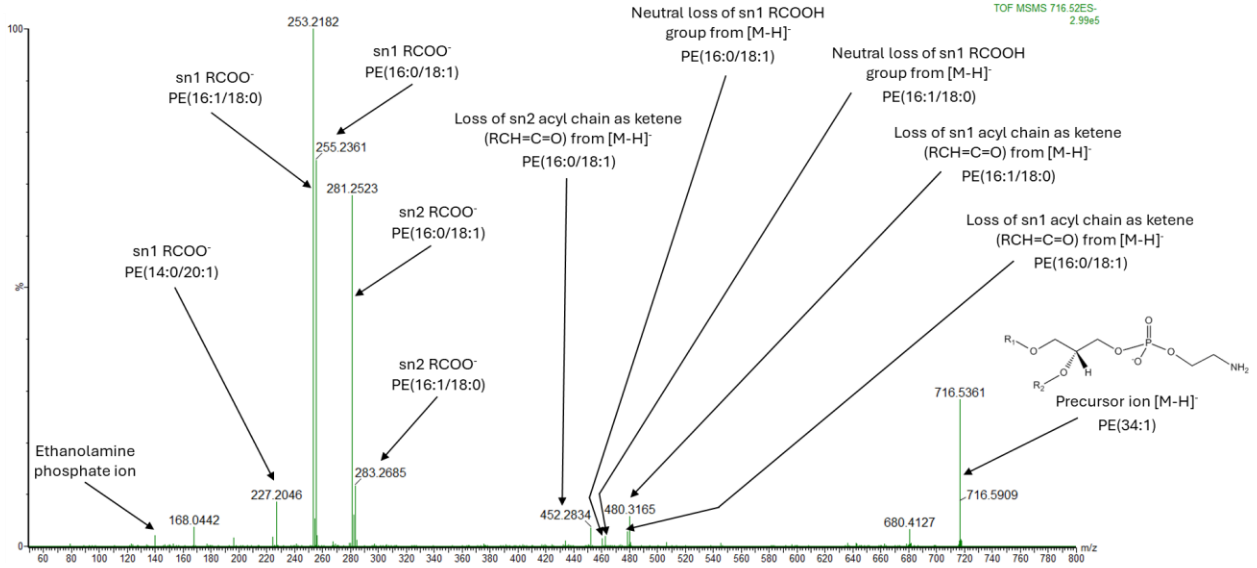

MS/MS spectrum of *m*/*z* 722.513 corresponding to PE(O-36:5) and PE(P-36:4).

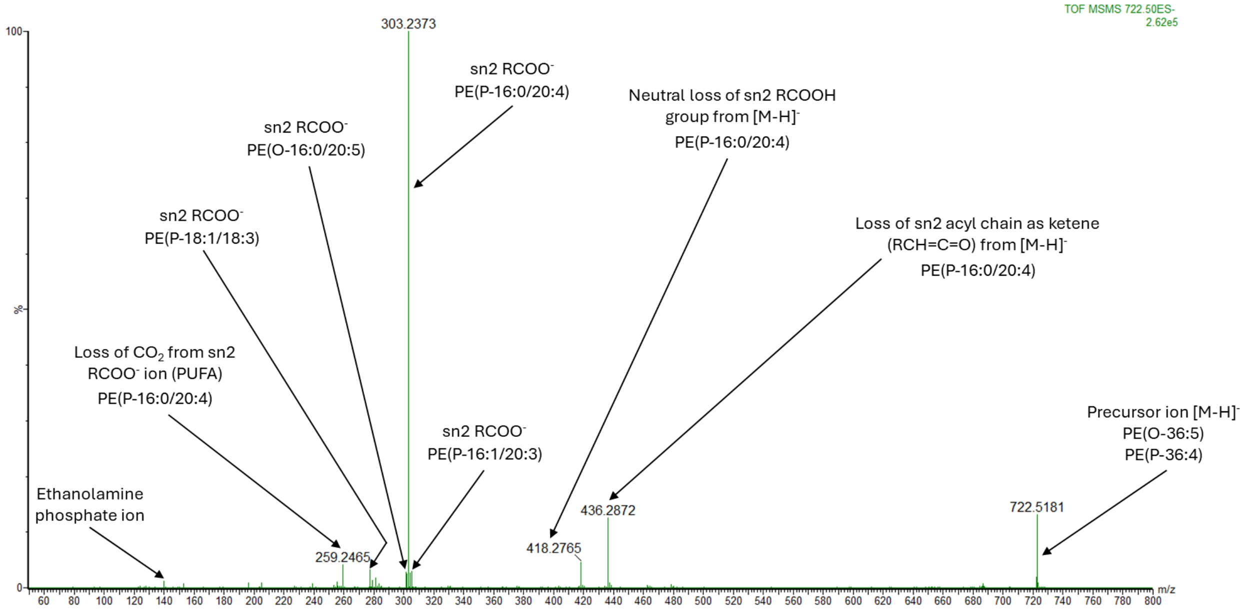

MS/MS spectrum of *m/z* 723.497 corresponding to PA(38:4).

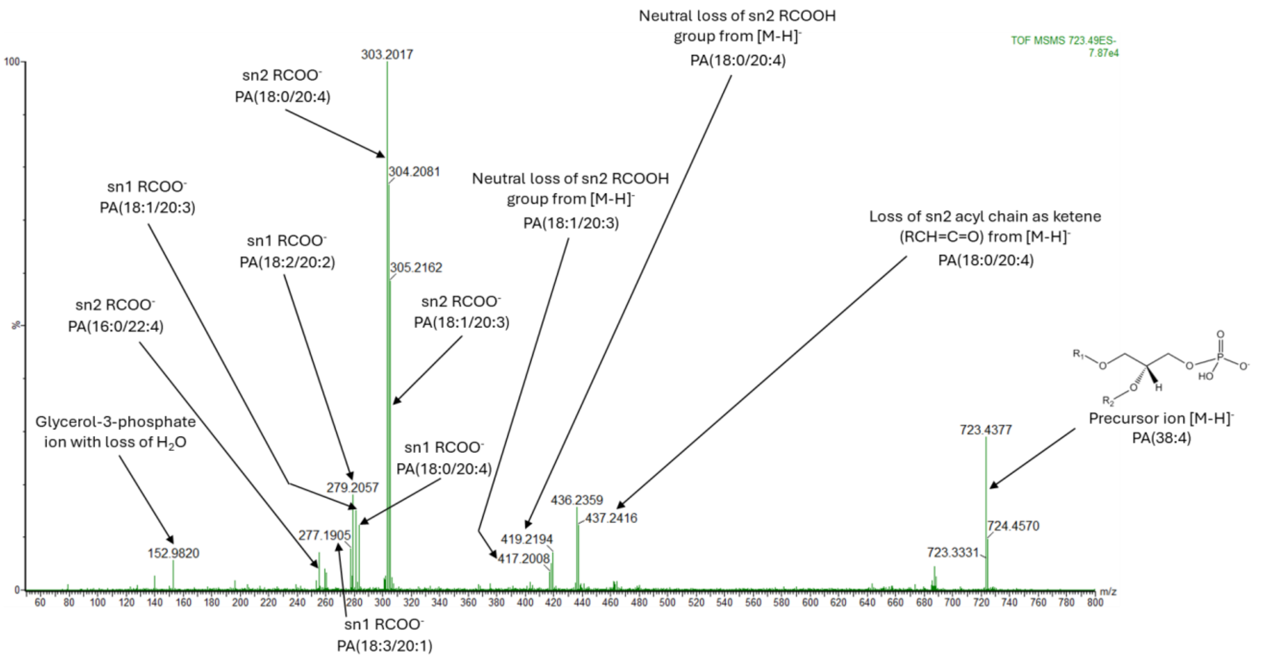

MS/MS spectrum of *m/z* 725.513 corresponding to PA(38:3).

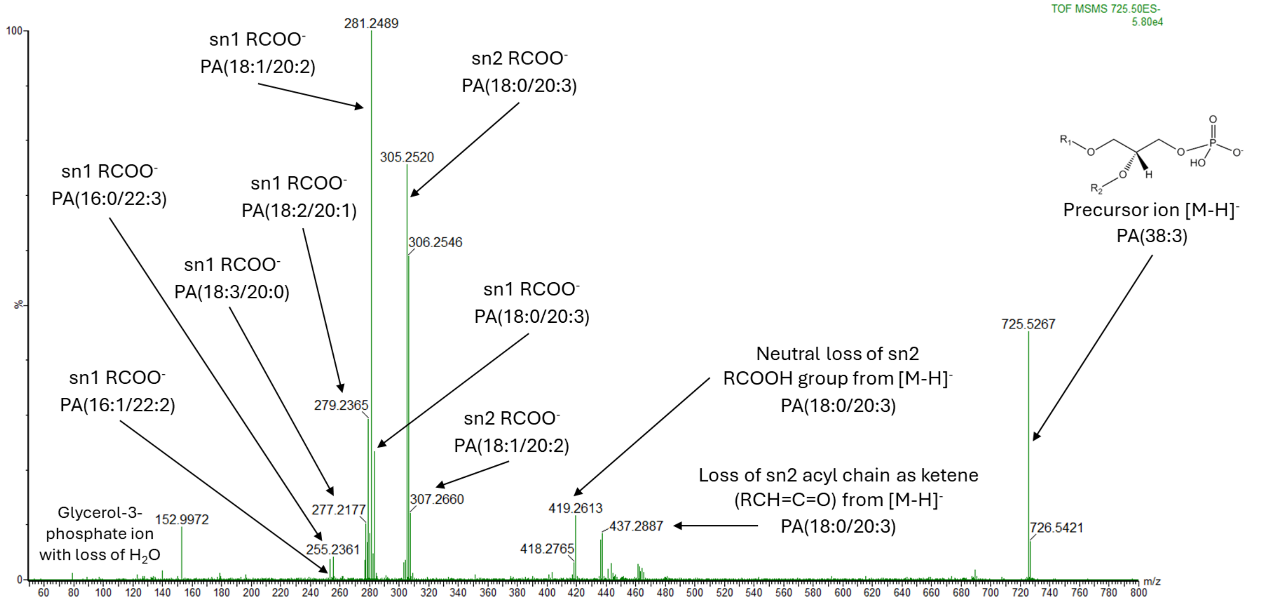

MS/MS spectrum of *m*/*z* 726.544 corresponding to PE(O-36:3) and PE(P-36:2).

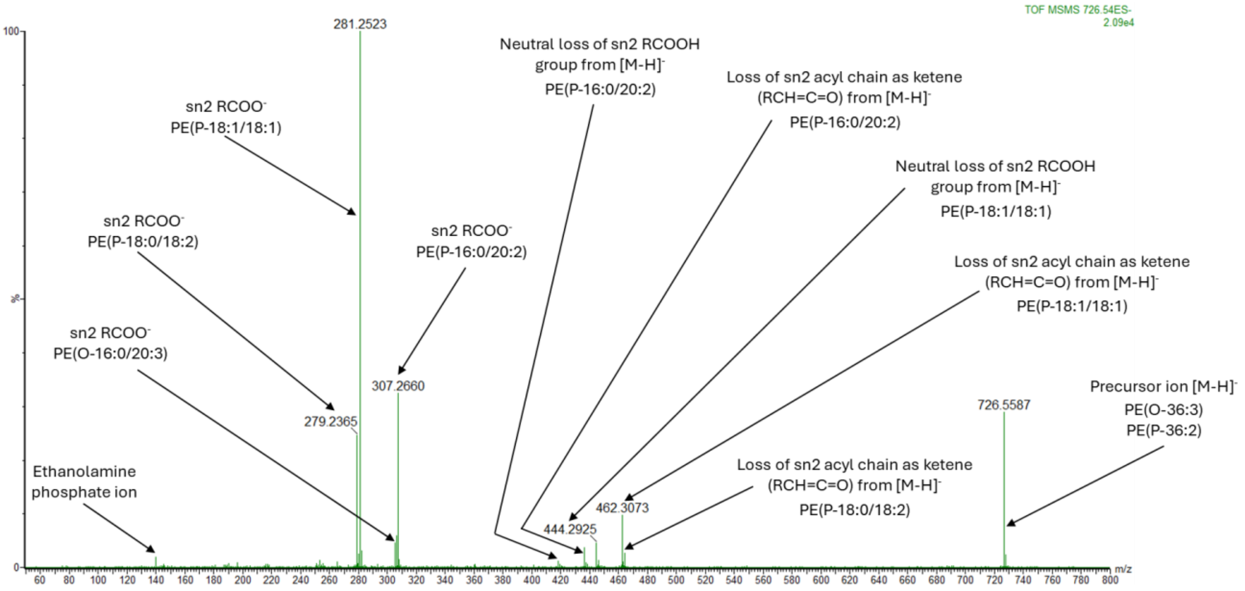

MS/MS spectrum of *m*/*z* 727.528 corresponding to PA(38:2).

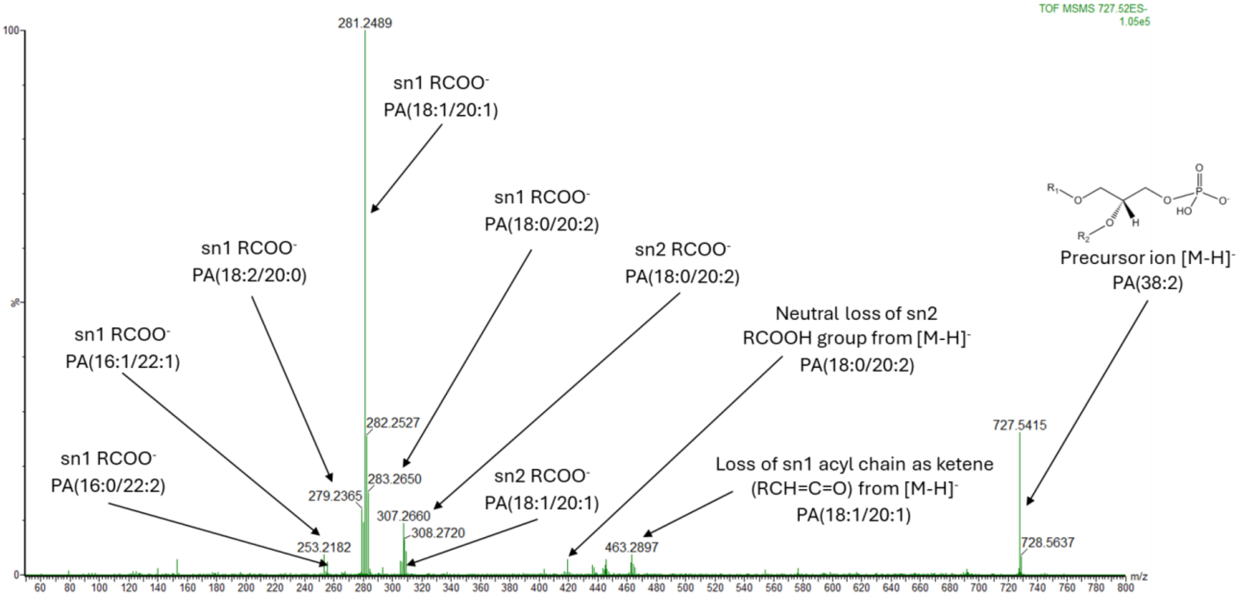

MS/MS spectrum of *m*/*z* 728.560 corresponding to PE(P-36:1).

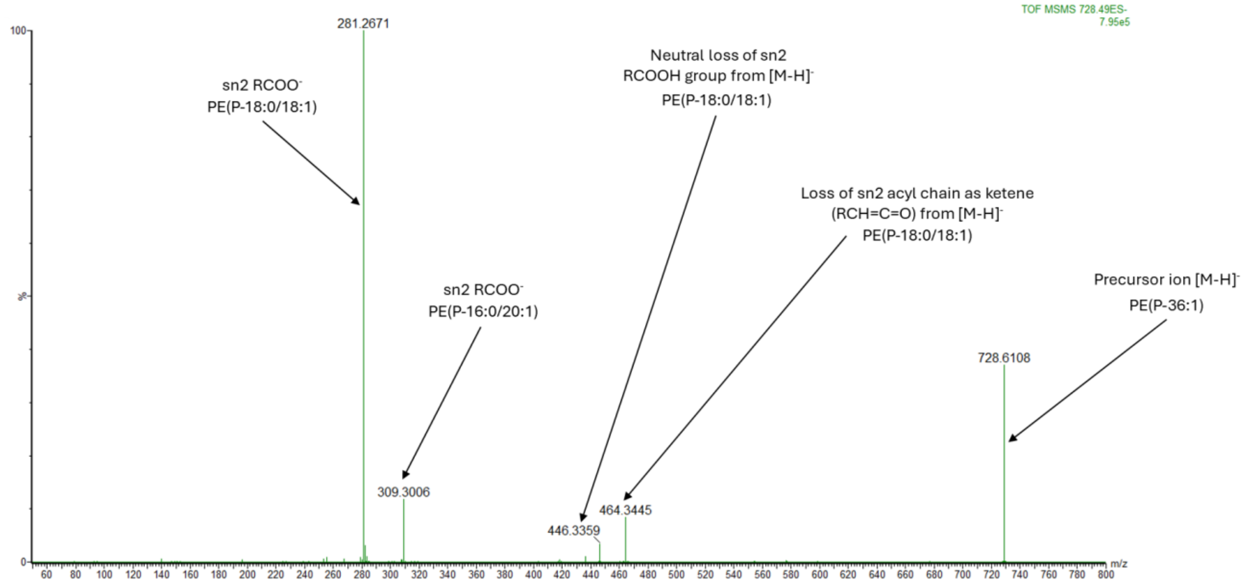

MS/MS spectrum of *m*/*z* 729.544 corresponding to PA(38:1).

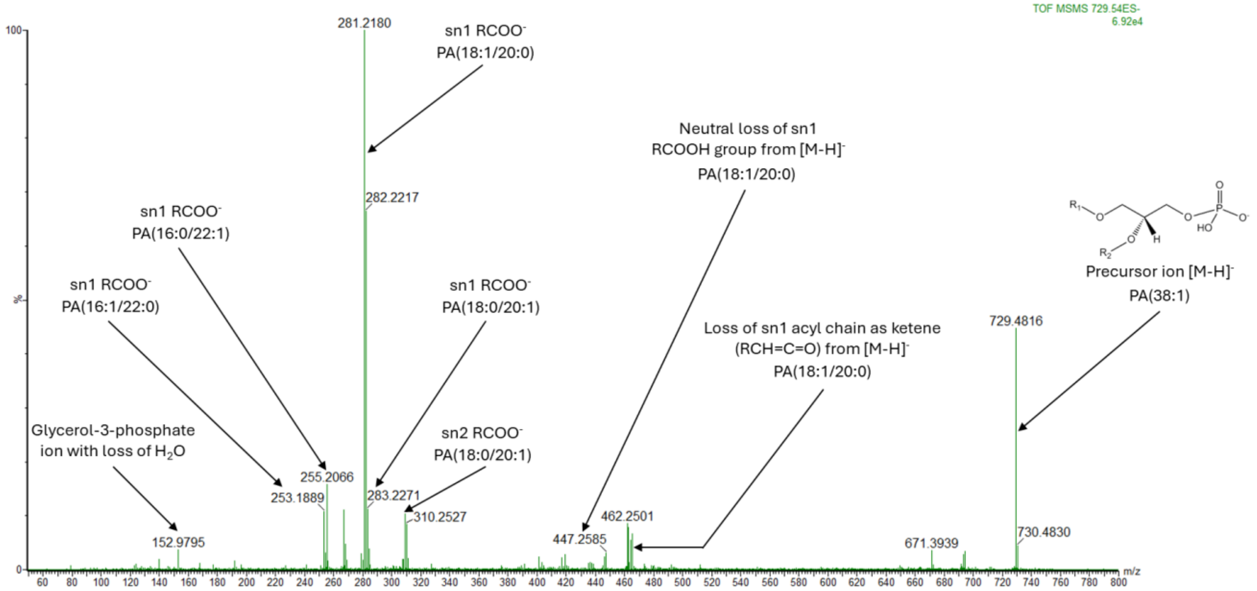

MS/MS spectrum of *m/z* 733.5 corresponding to PE(34:2) and PA(36:3) (*m/z* 733.458), and PE(O-38:5) and PE(P-38:4) (*m/z* 733.518).

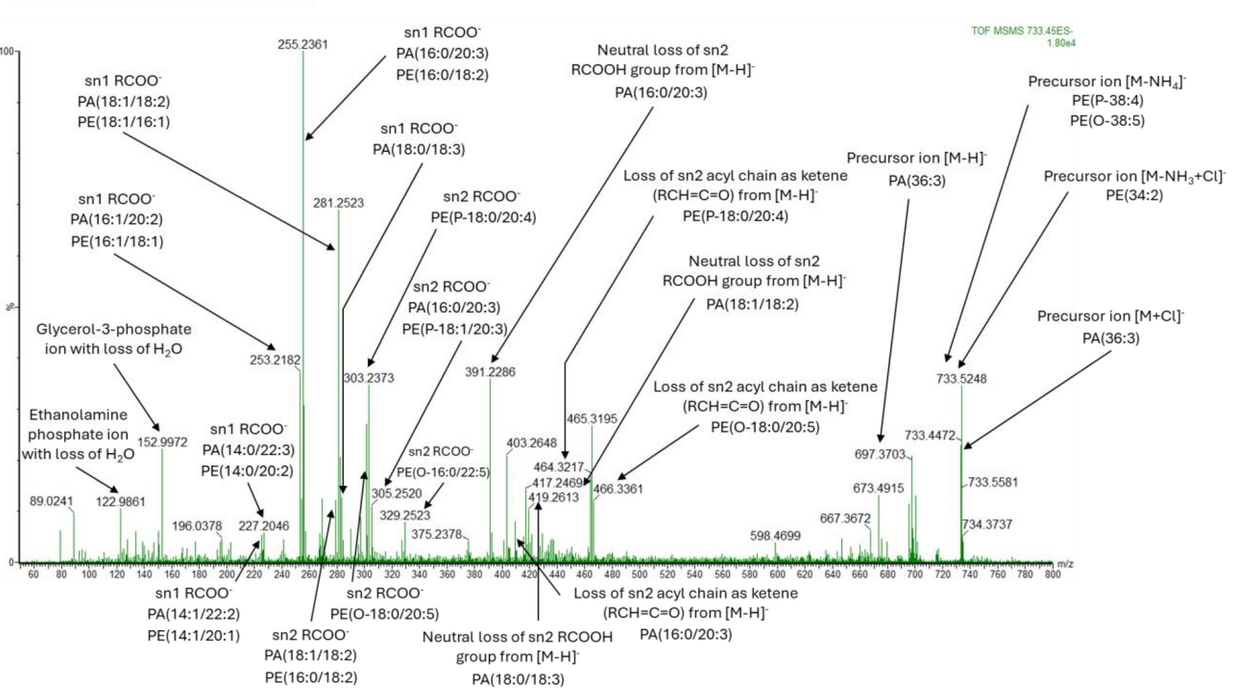

MS/MS spectrum of *m/z* 735.4 corresponding to PE(34:1) and PA(36:2) (*m/z* 735.474), and PS(O-36:5) and PS(P-36:4) (*m/z* 735.461).

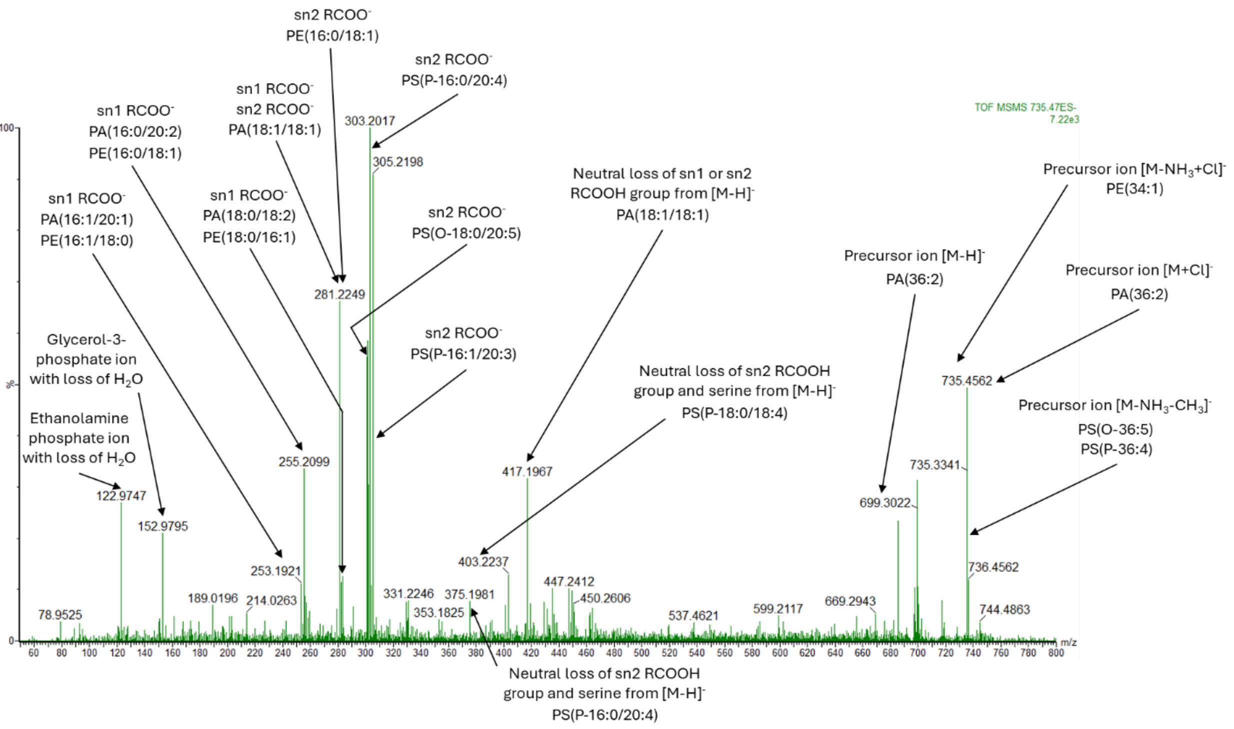

MS/MS spectrum of *m/z* 737.5 corresponding to PA(36:1) (*m/z* 737.489) and PA(40:4) (*m/z* 737.513).

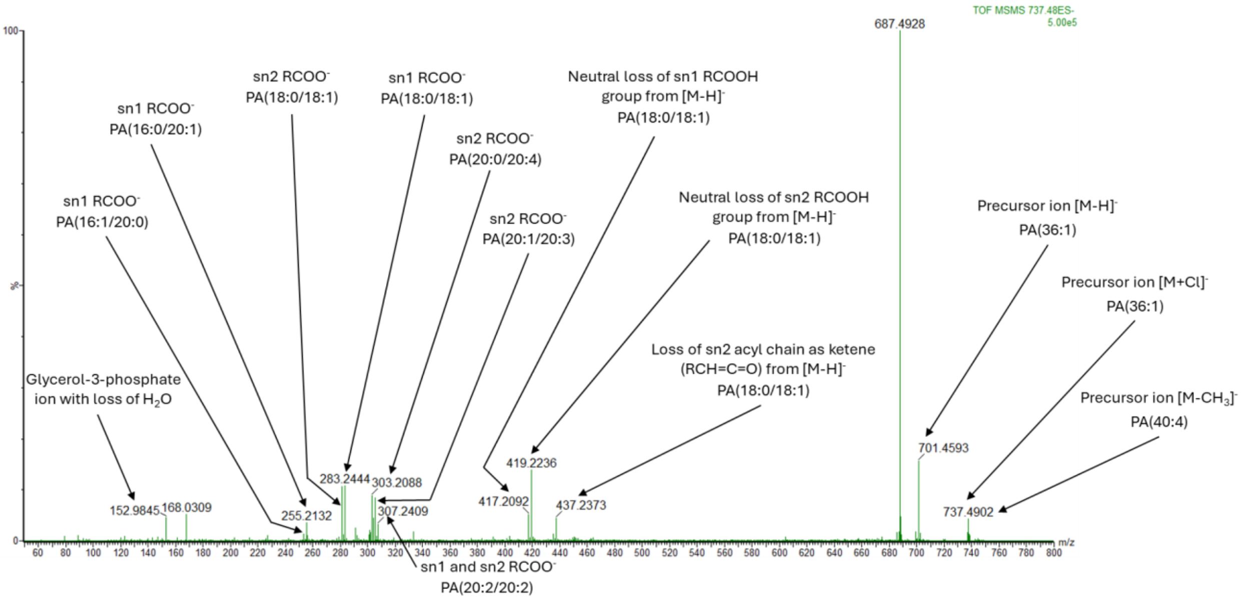

MS/MS spectrum of *m/z* 740.524 corresponding to PE(36:3).

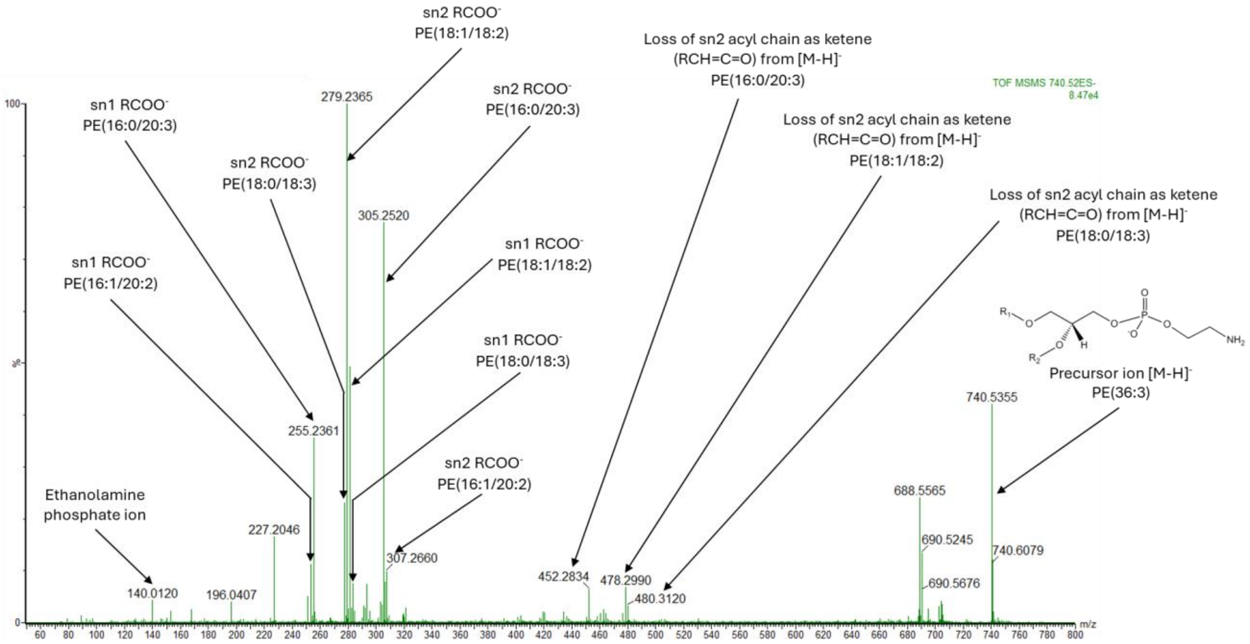

MS/MS spectrum of *m*/*z* 742.539 corresponding to PE(36:2).

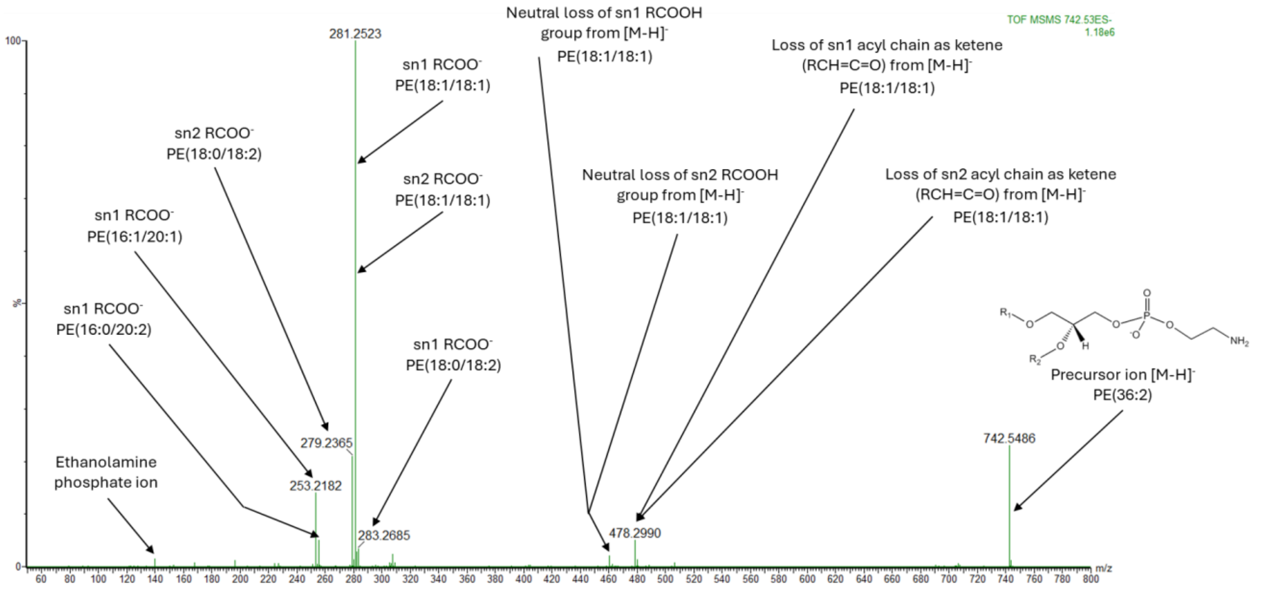

MS/MS spectrum of *m*/*z* 744.555 corresponding to PE(36:1).

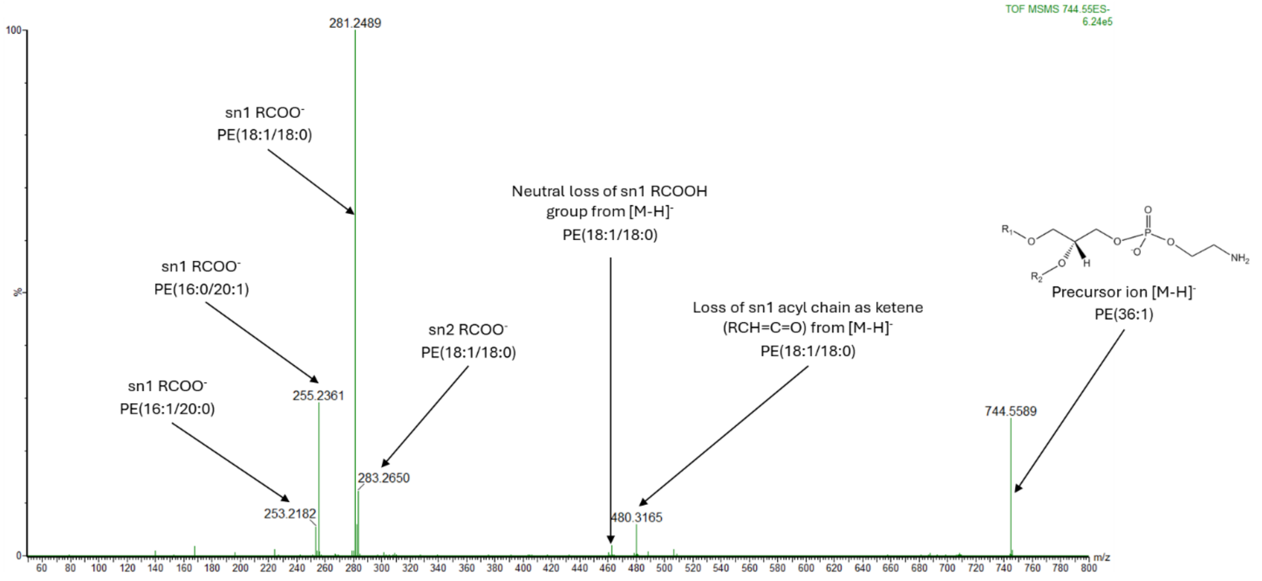

MS/MS spectrum of *m/z* 747.497 corresponding to PA(40:6) and PE(38:5).

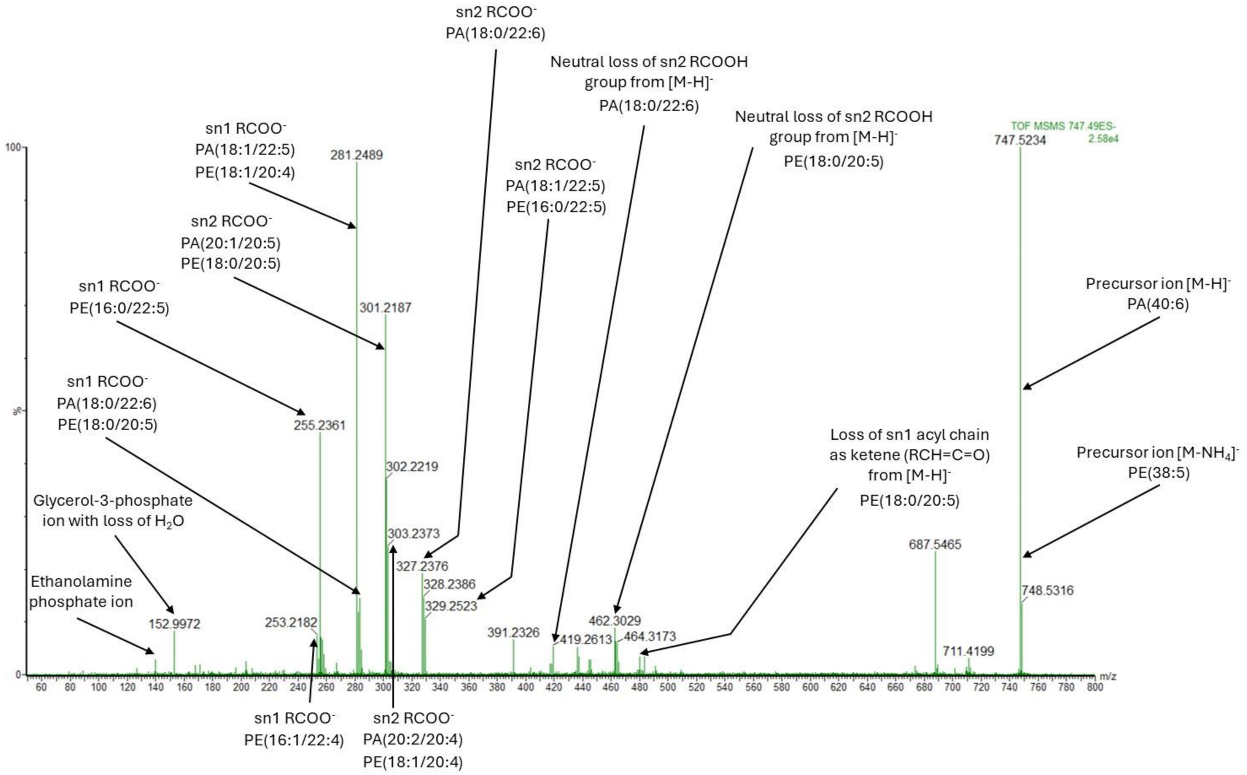

MS/MS spectrum of *m/z* 748.529 corresponding to PE(O-38:6) and PE(P-38:5).

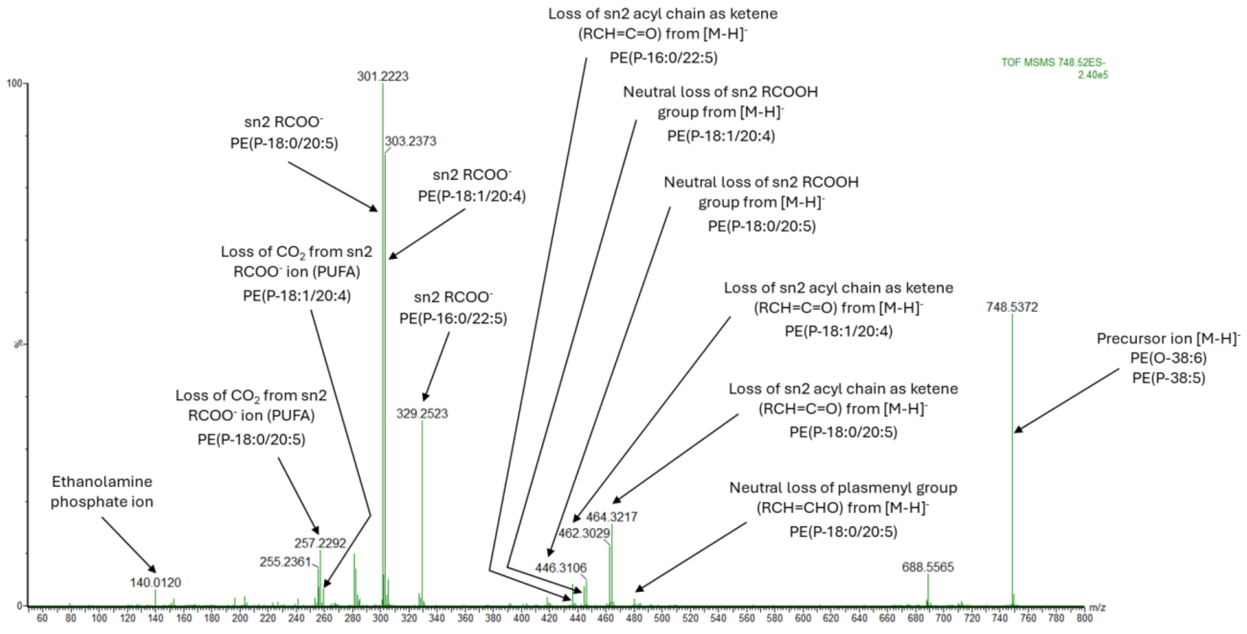

MS/MS spectrum of *m*/*z* 749.513 corresponding to PA(40:5) and PE(38:4).

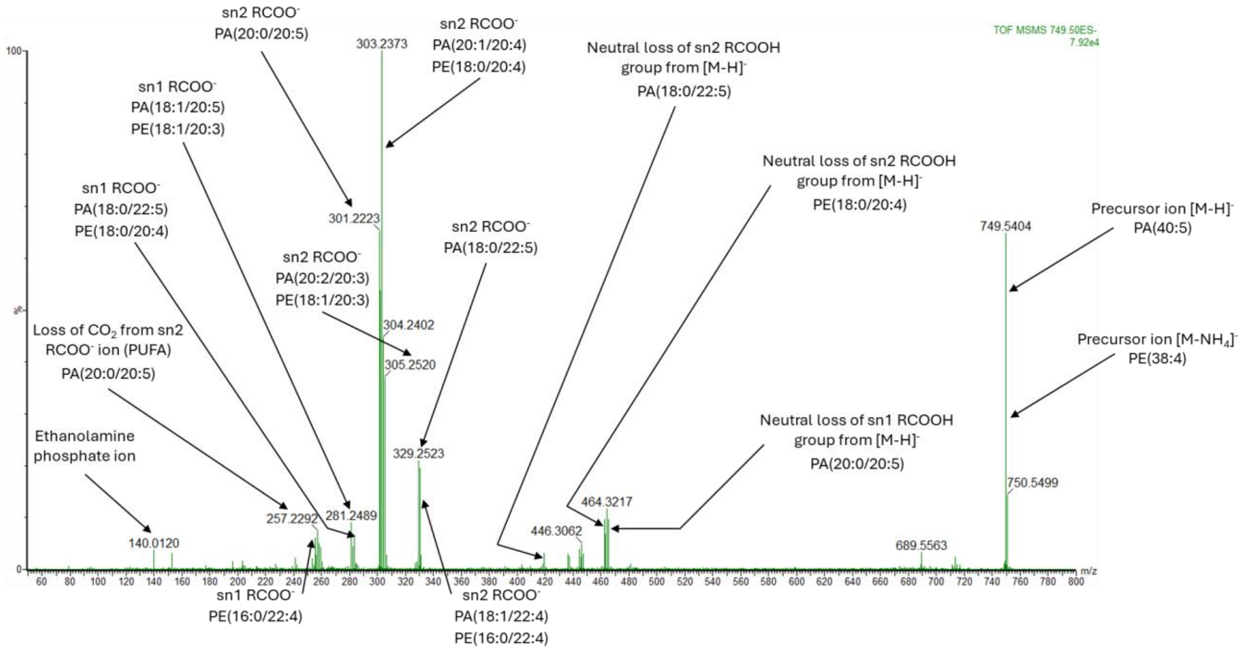

MS/MS spectrum of *m*/*z* 750.544 corresponding to PE(O-38:5) and PE(P-38:4).

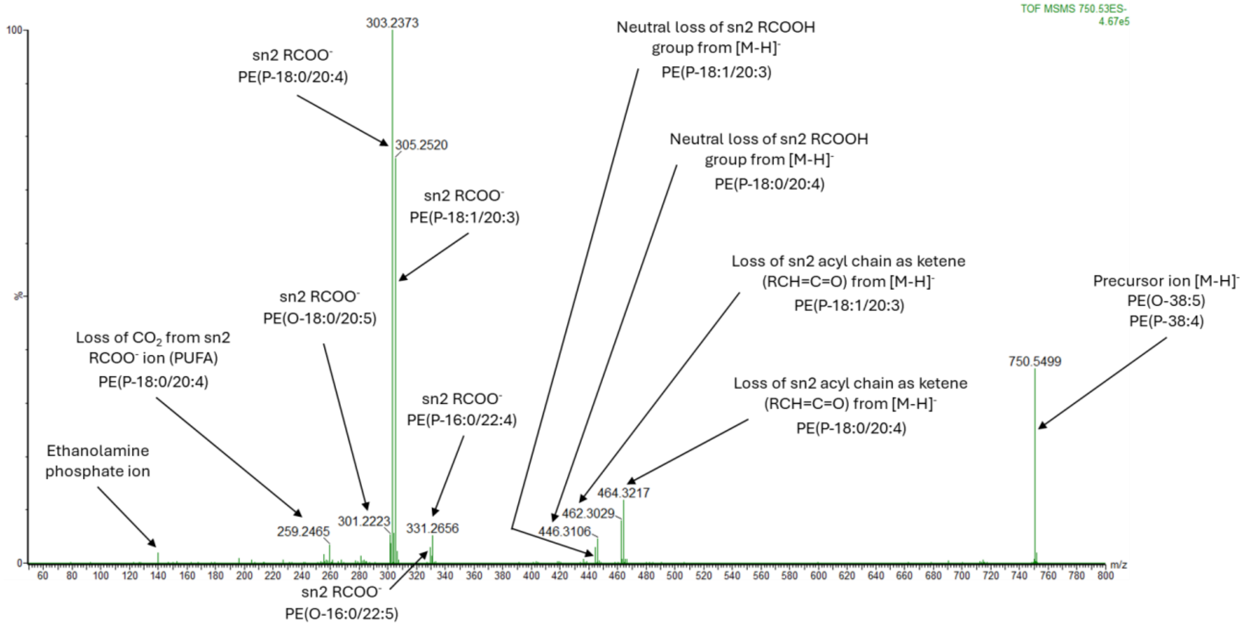

MS/MS spectrum of *m*/*z* 751.528 corresponding to PA(40:4) and PE(38:3).

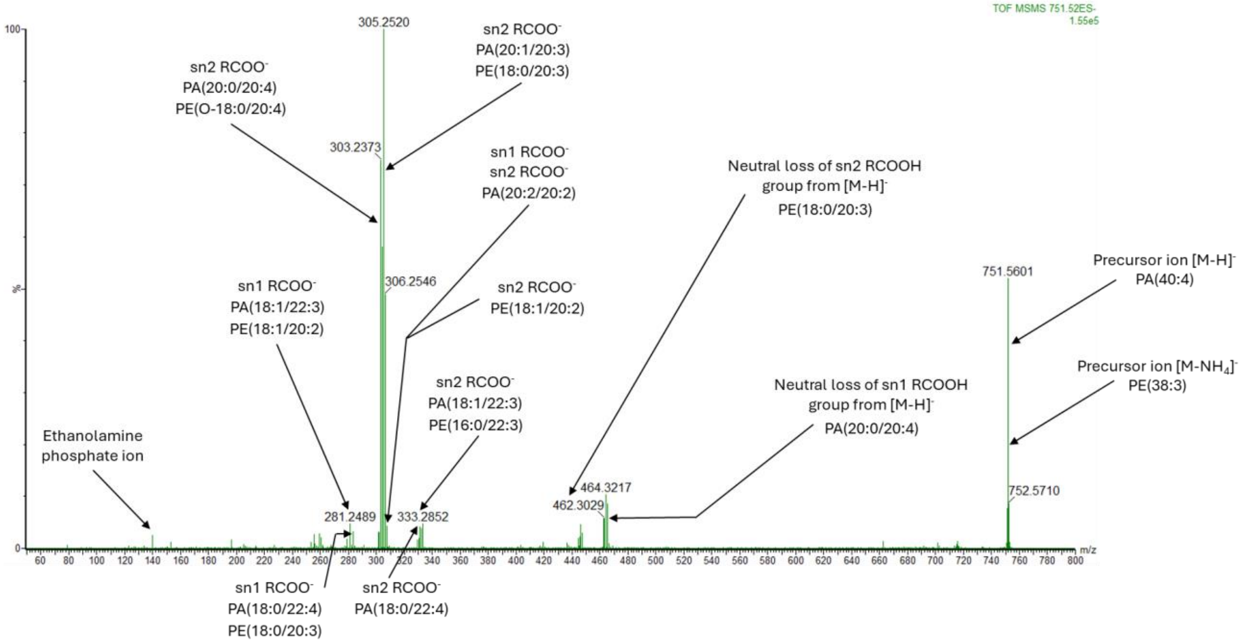

MS/MS spectrum of *m*/*z* 752.560 corresponding to PE(O-38:4) and PE(P-38:3).

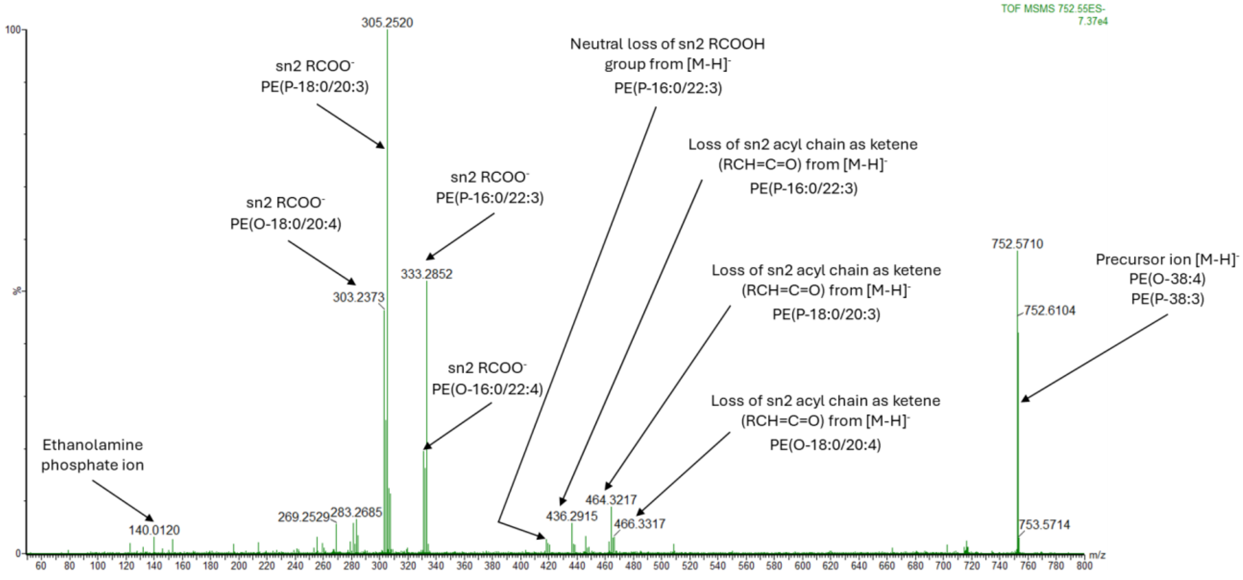

MS/MS spectrum of *m/z* 761.489 corresponding to PA(38:3), PS(O-38:6) and PS(P-38:5).

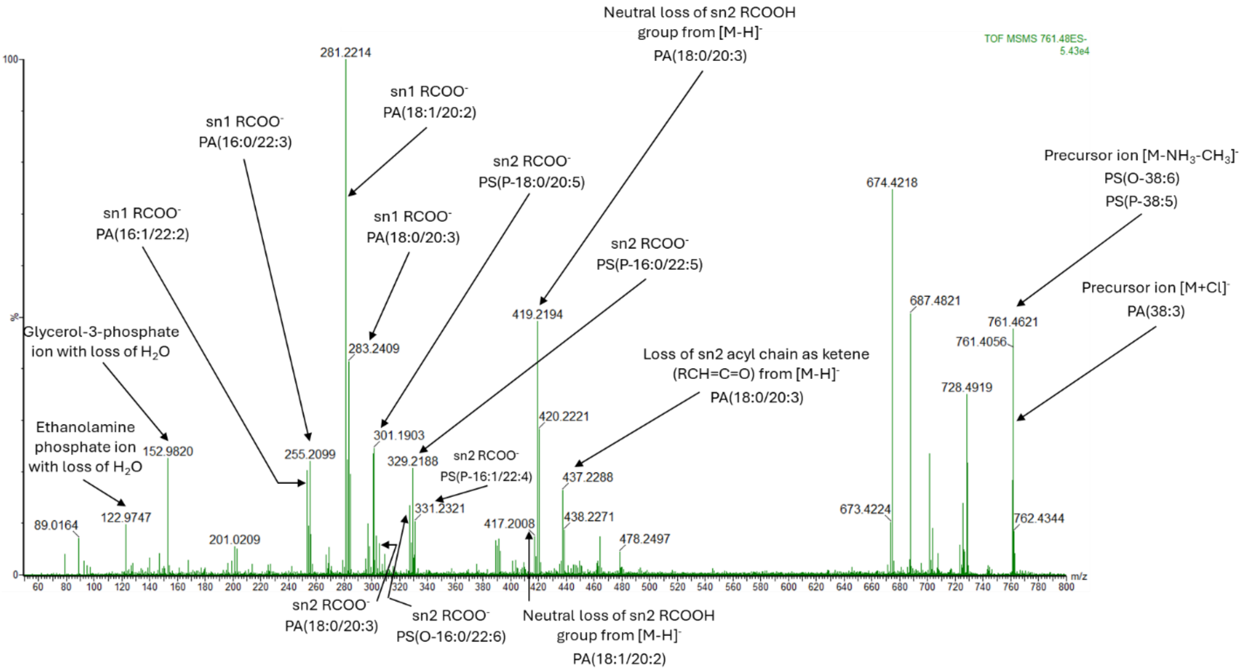

MS/MS spectrum of *m/z* 763.499 corresponding to PA(38:2), PS(O-38:5) and PS(P-38:4).

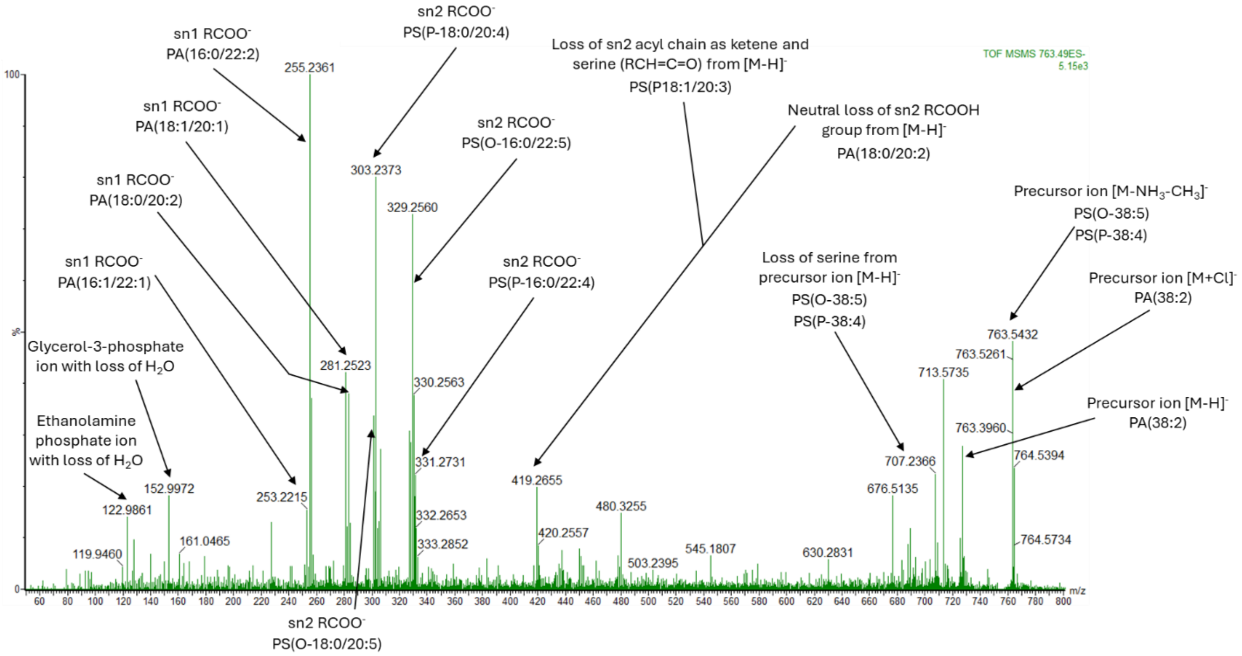

MS/MS spectrum of *m*/*z* 766.539 corresponding to PE(38:4).

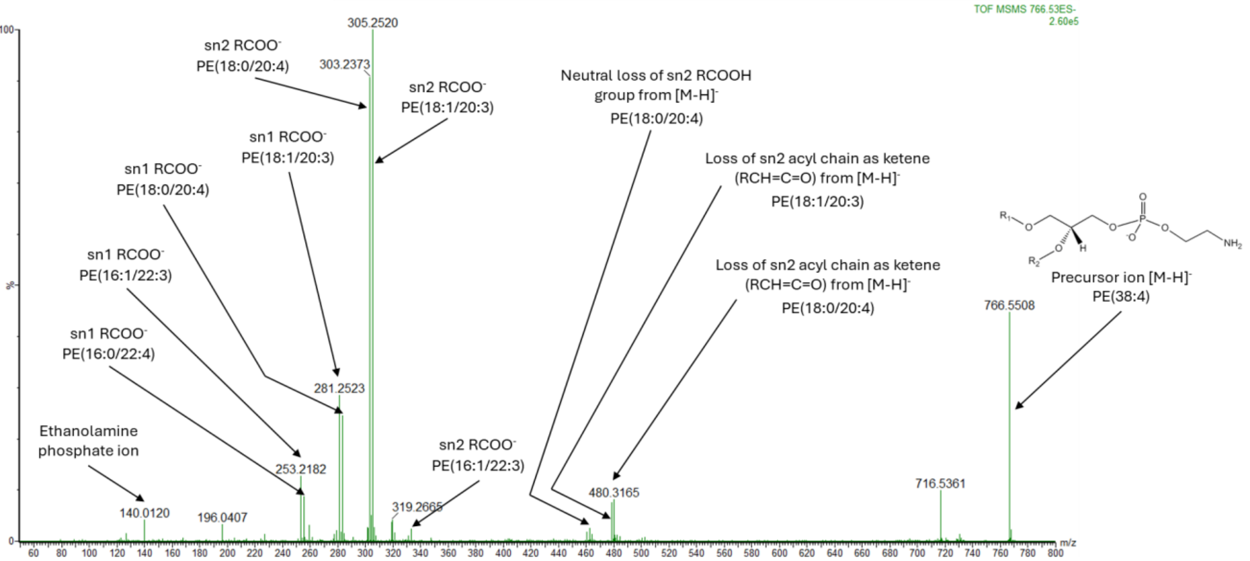

MS/MS spectrum of *m*/*z* 768.555 corresponding to PE(38:3).

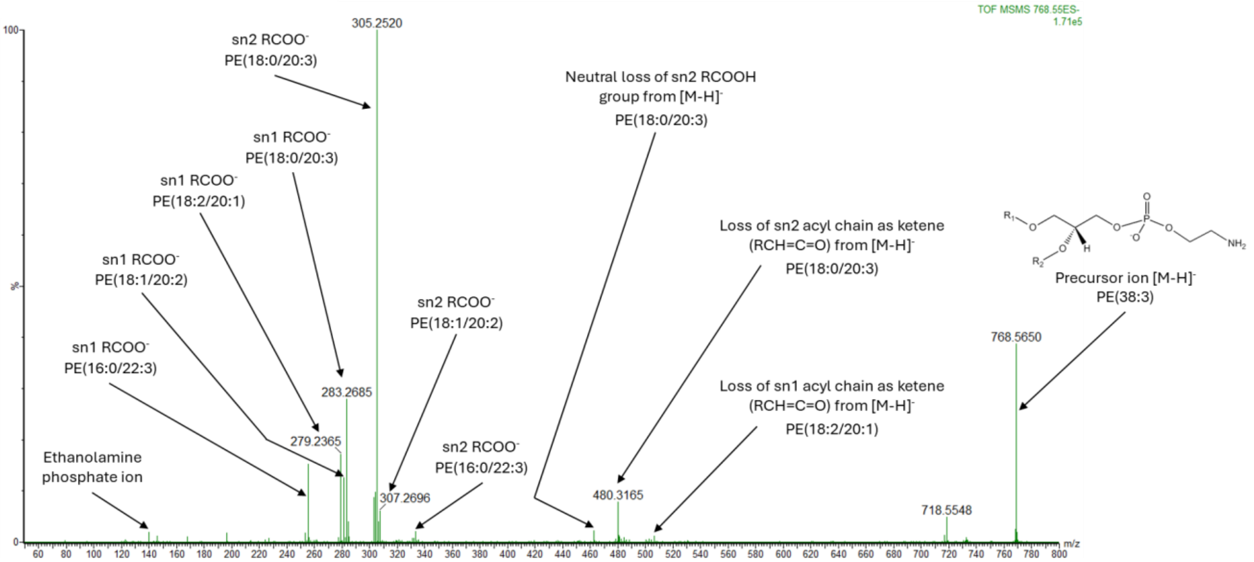

MS/MS spectrum of *m/z* 773.534 corresponding to PG(36:2).

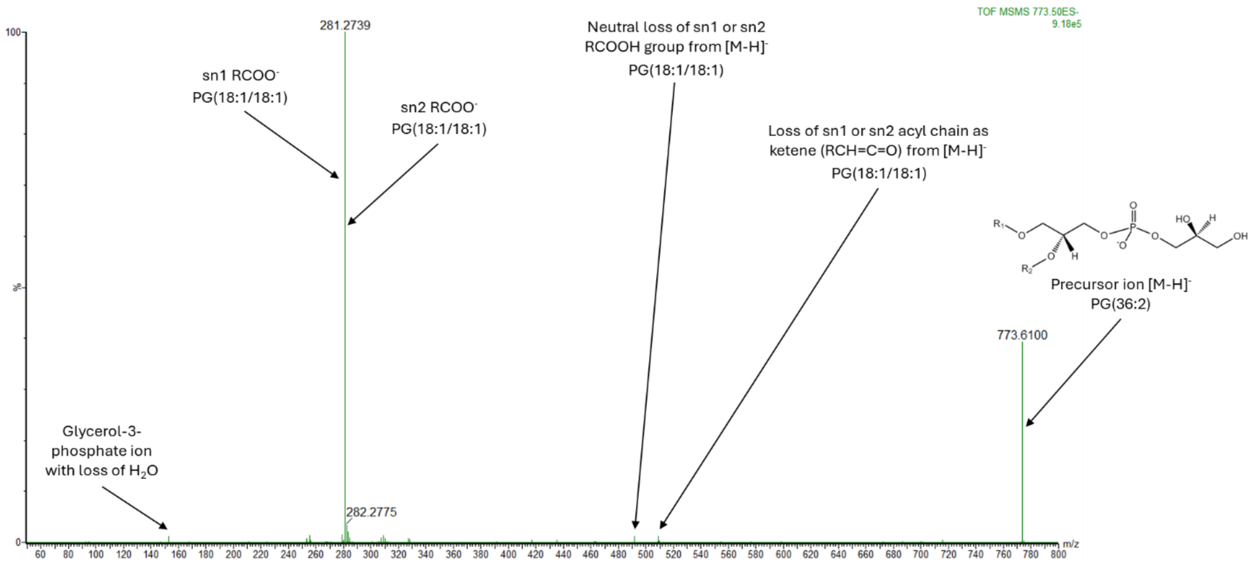

MS/MS spectrum of *m/z* 774.544 corresponding to PE(P-40:6) and PS(36:1).

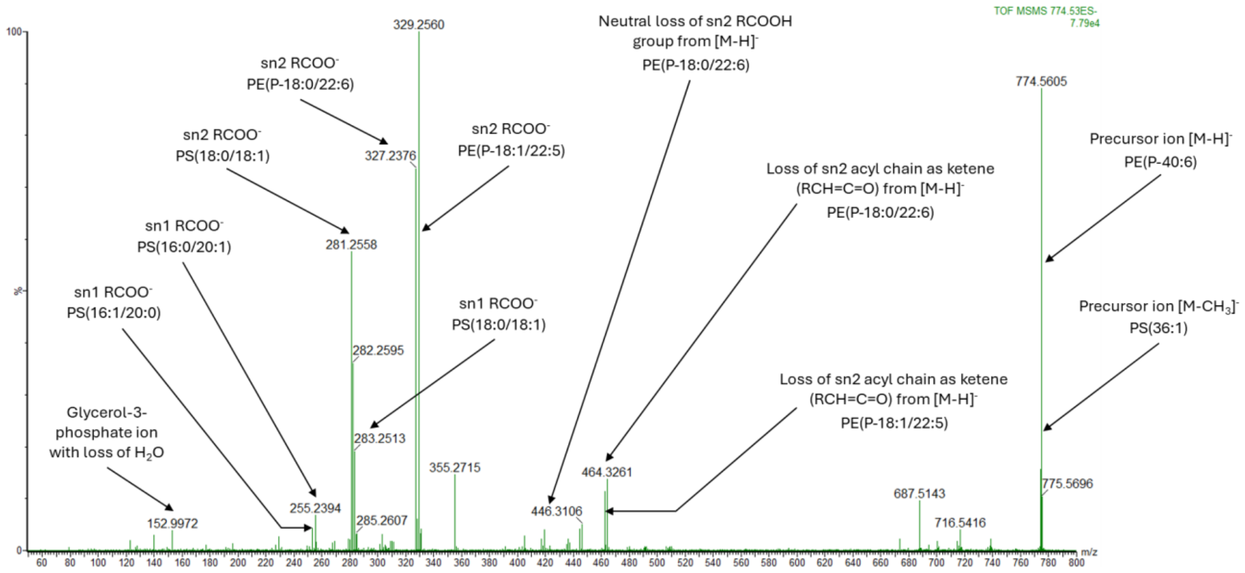

MS/MS spectrum of *m*/*z* 778.576 corresponding to PE(O-40:5) and PE(P-40:4).

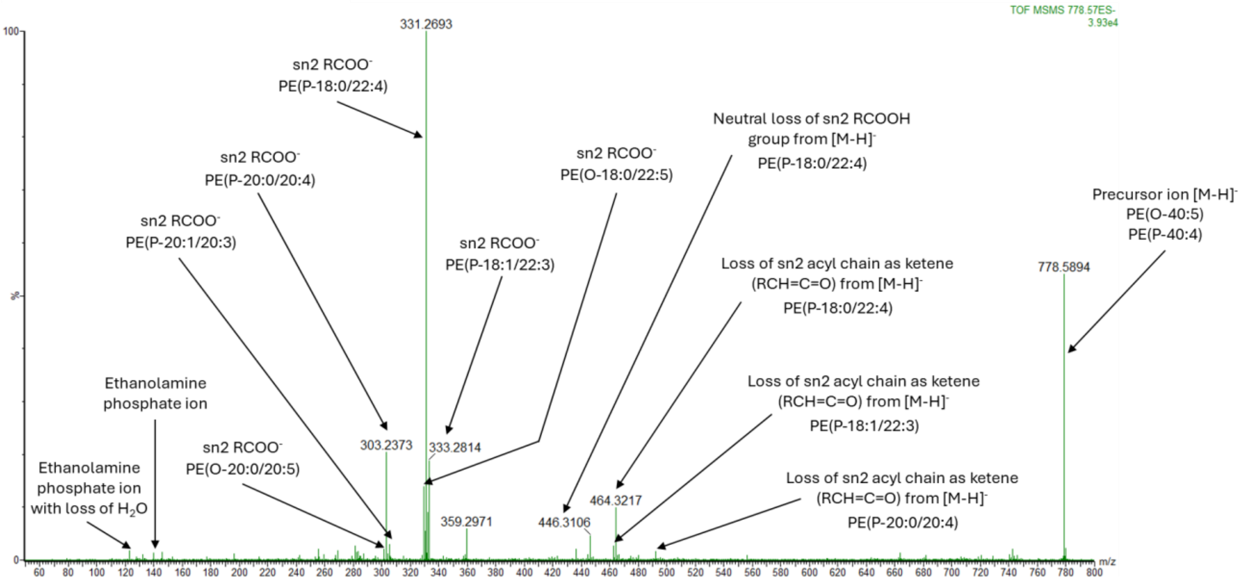

MS/MS spectrum of *m*/*z* 788.545 corresponding to PS(36:1).

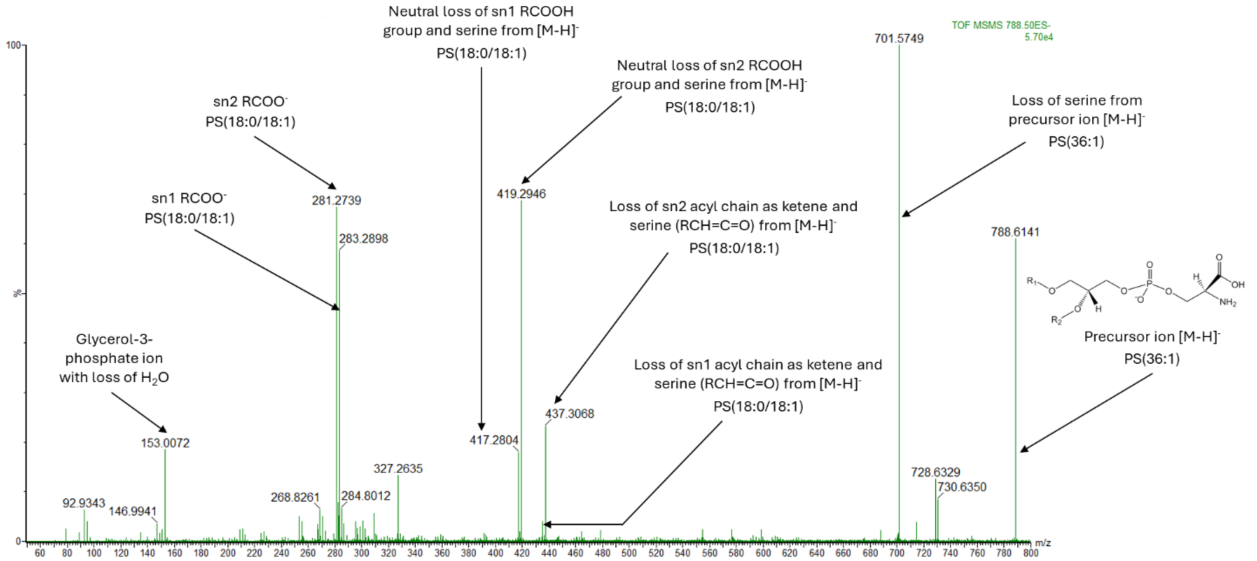

MS/MS spectrum of *m*/*z* 794.571 corresponding to PE(40:4) and PC(38:4).

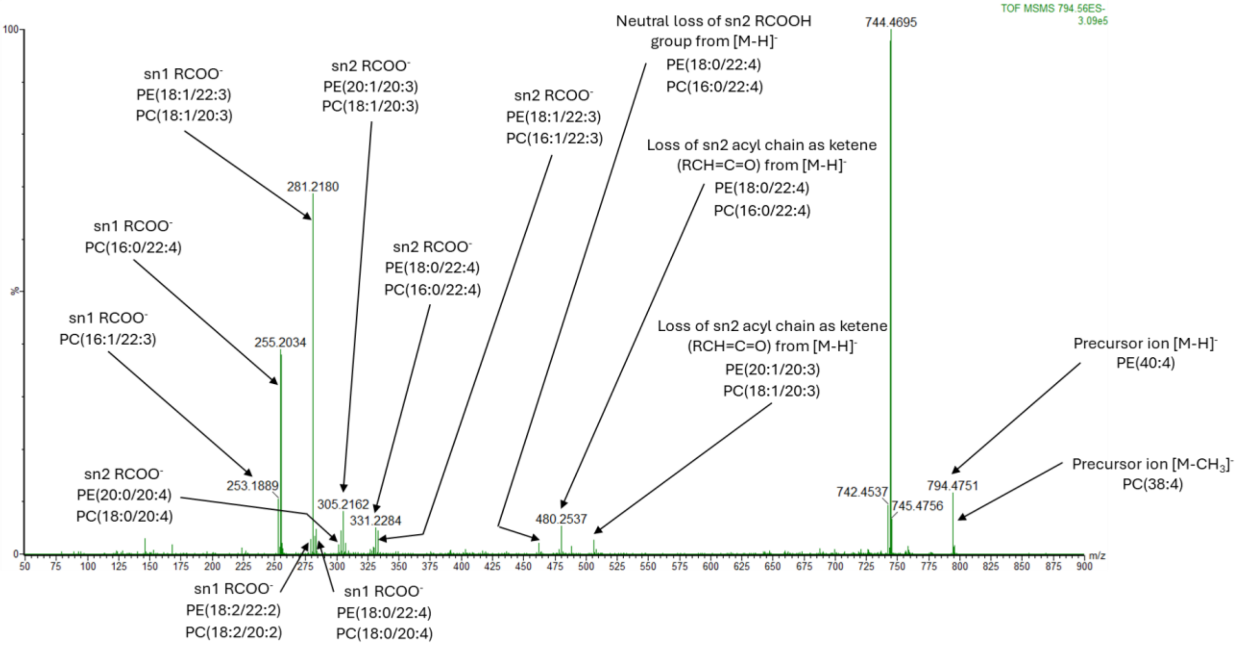

MS/MS spectrum of *m*/*z* 810.529 corresponding to PS(38:4).

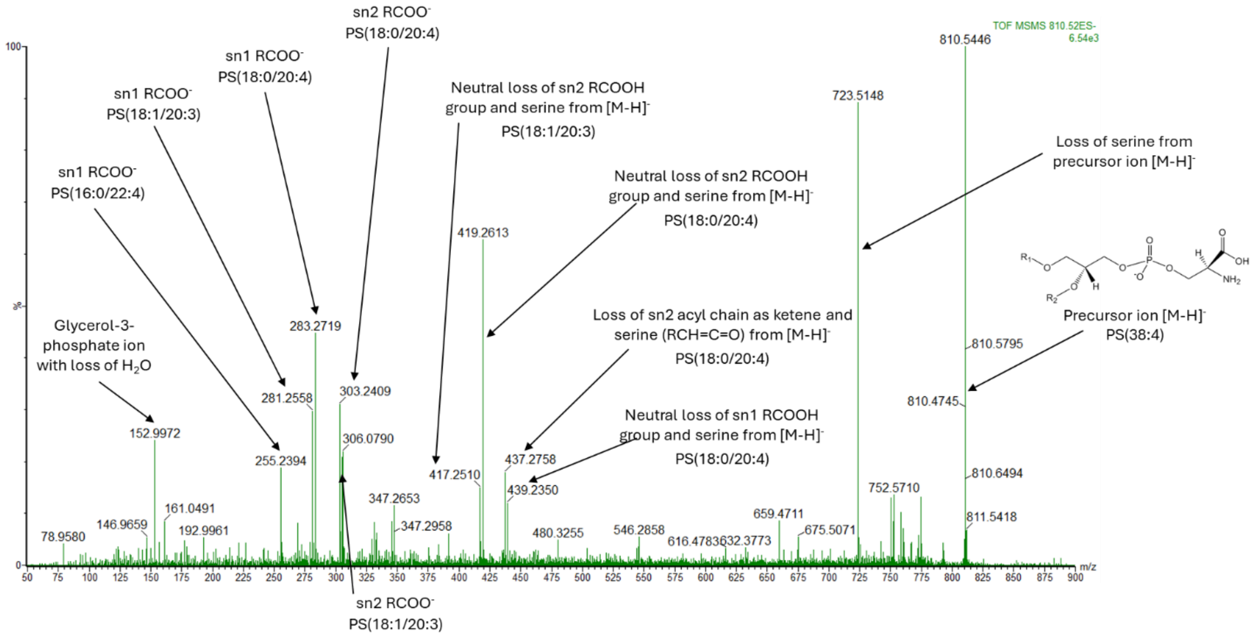

MS/MS spectrum of *m*/*z* 812.545 corresponding to PS(38:3).

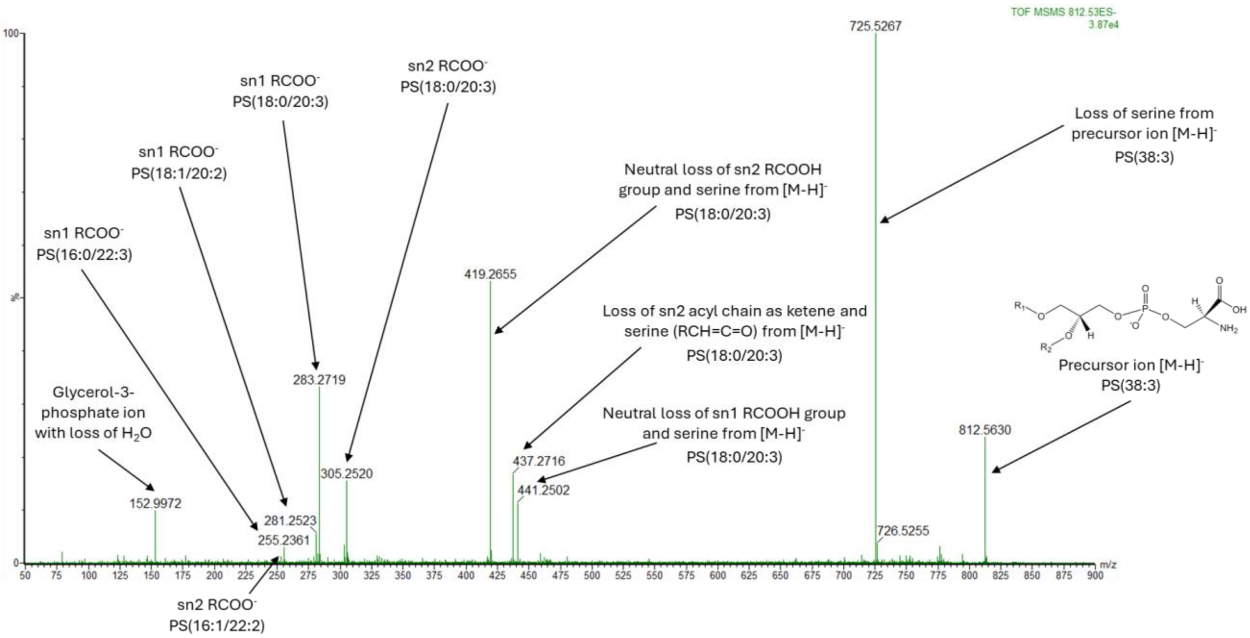

MS/MS spectrum of *m*/*z* 819.518 corresponding to PG(40:7).

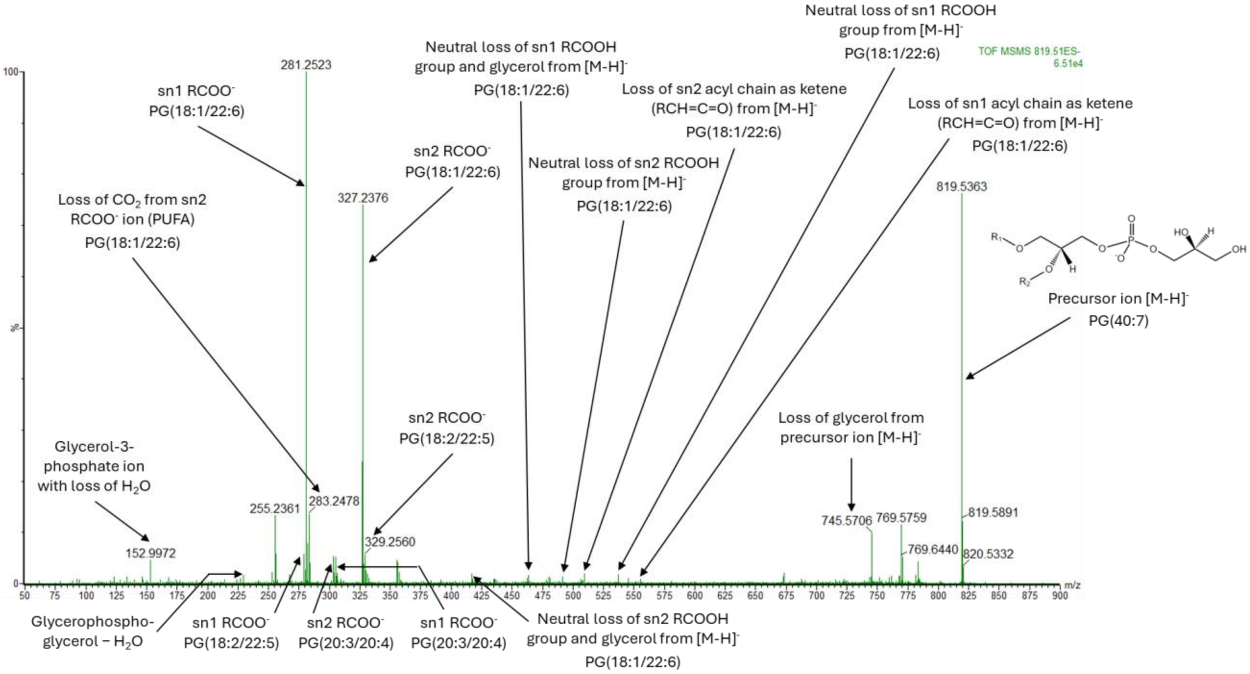

MS/MS spectrum of *m*/*z* 833.519 corresponding to PI(34:2).

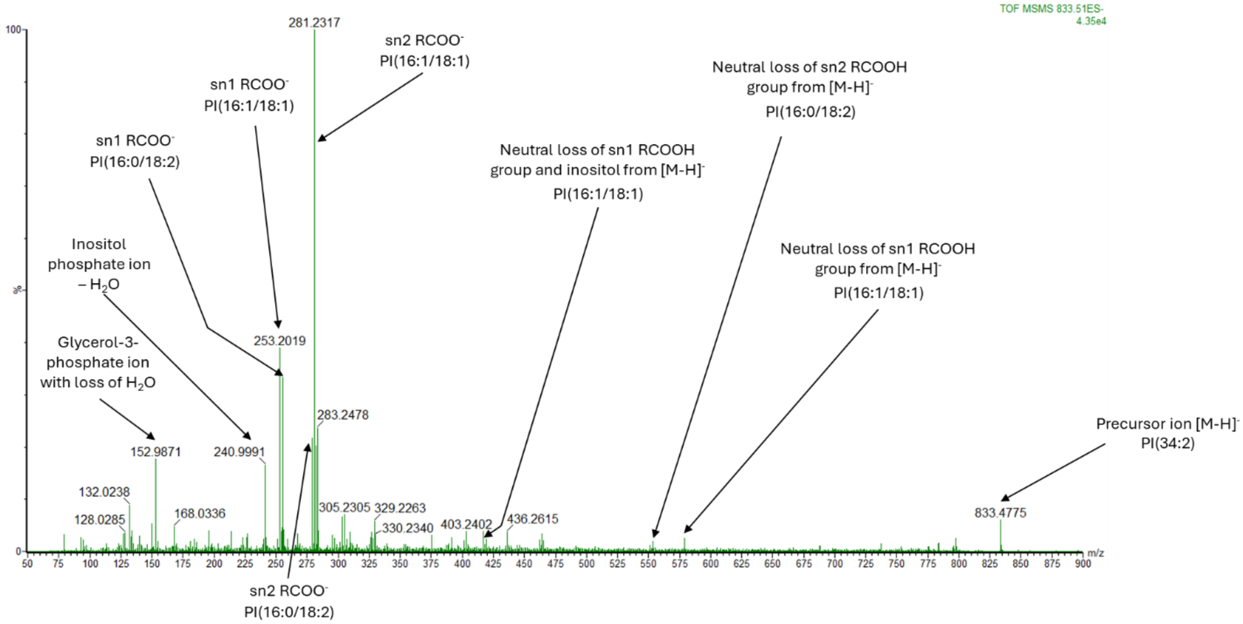

MS/MS spectrum of *m*/*z* 835.534 corresponding to PI(34:1).

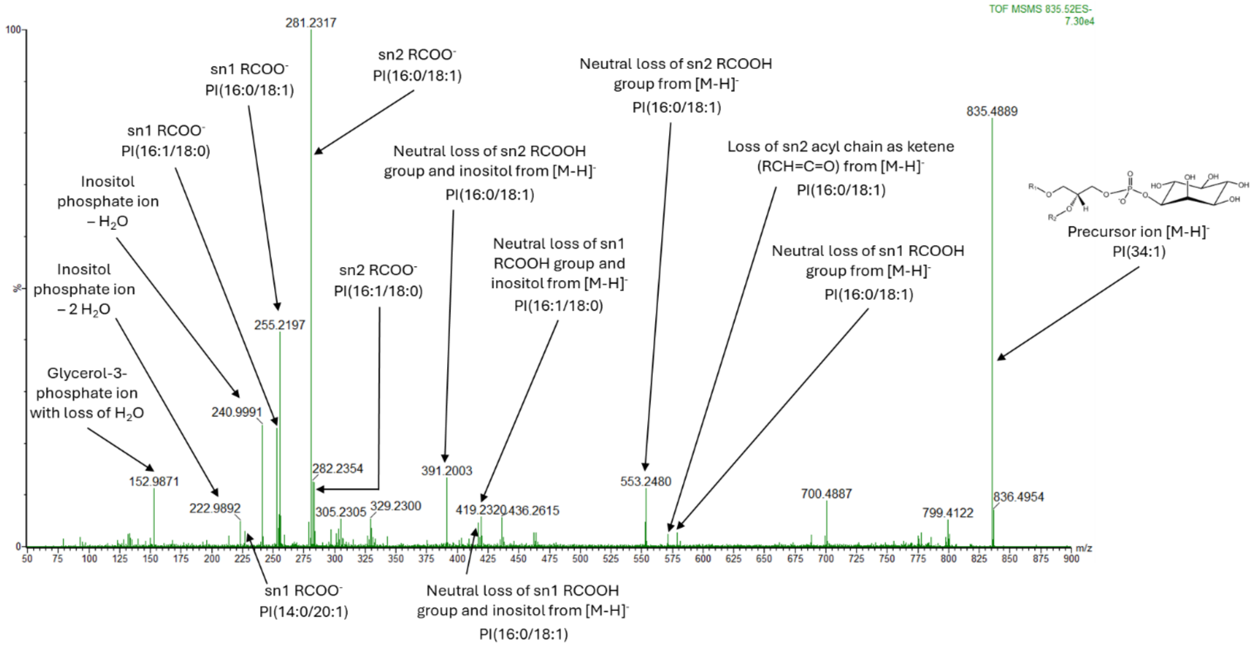

MS/MS spectrum of *m*/*z* 838.560 corresponding to PS(40:4) and PC(36:5).

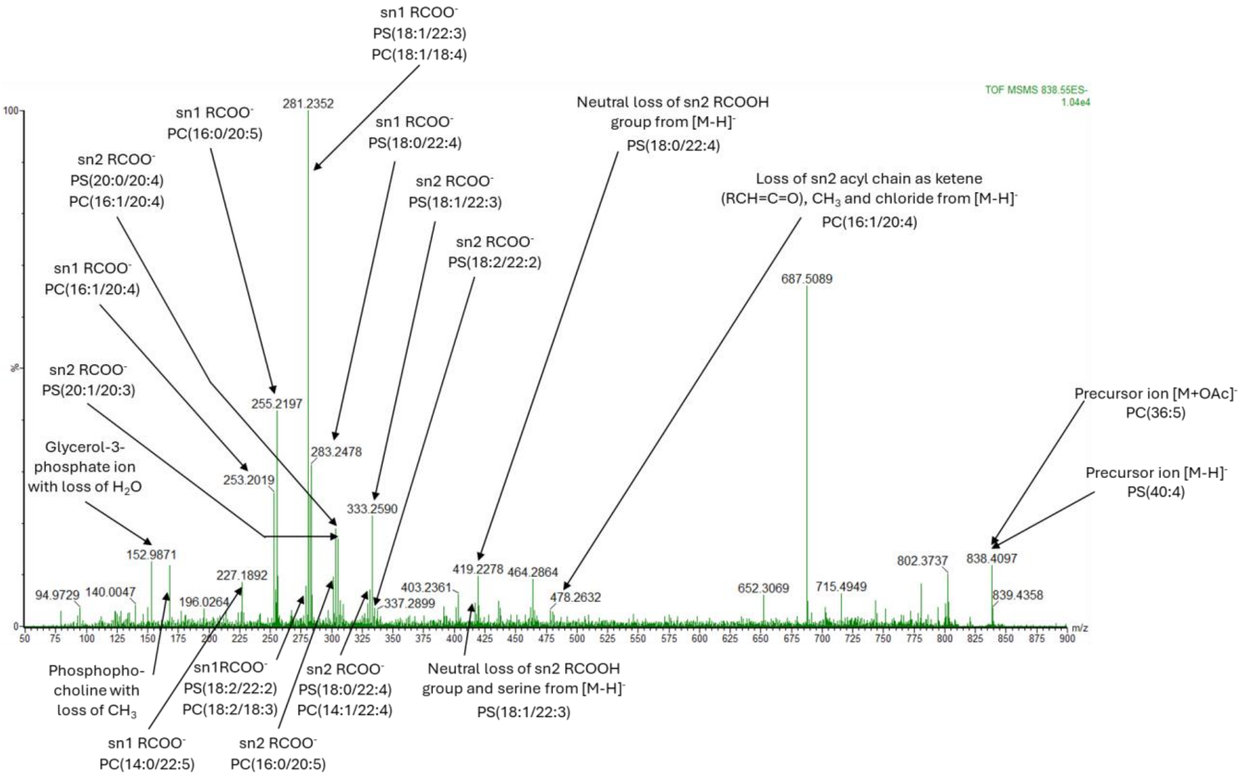

MS/MS spectrum of *m*/*z* 857.519 corresponding to PI(36:4).

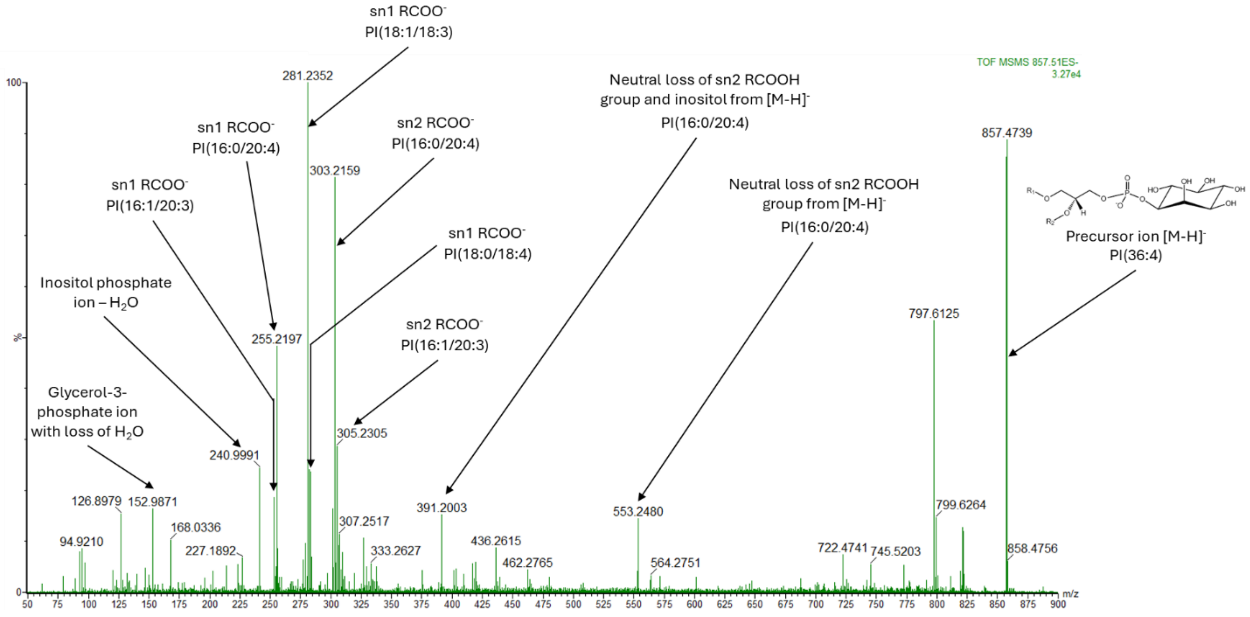

MS/MS spectrum of *m*/*z* 861.550 corresponding to PI(36:2) and PG(40:4).

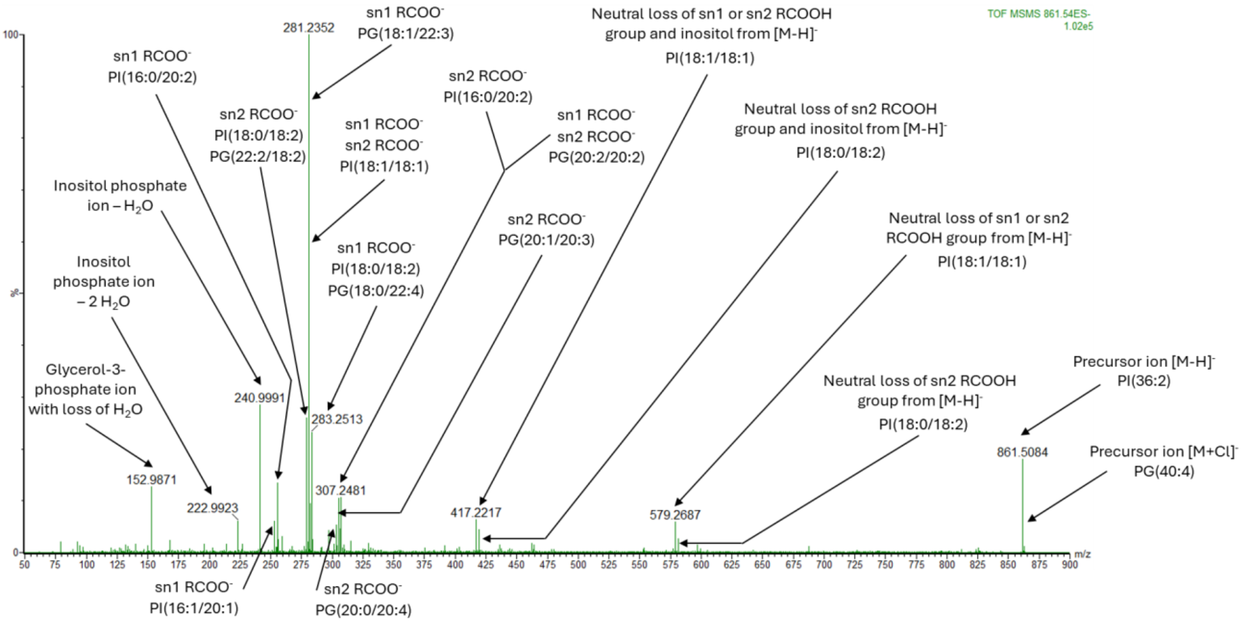

MS/MS spectrum of *m*/*z* 863.566 corresponding to PI(36:1) and PG(40:3).

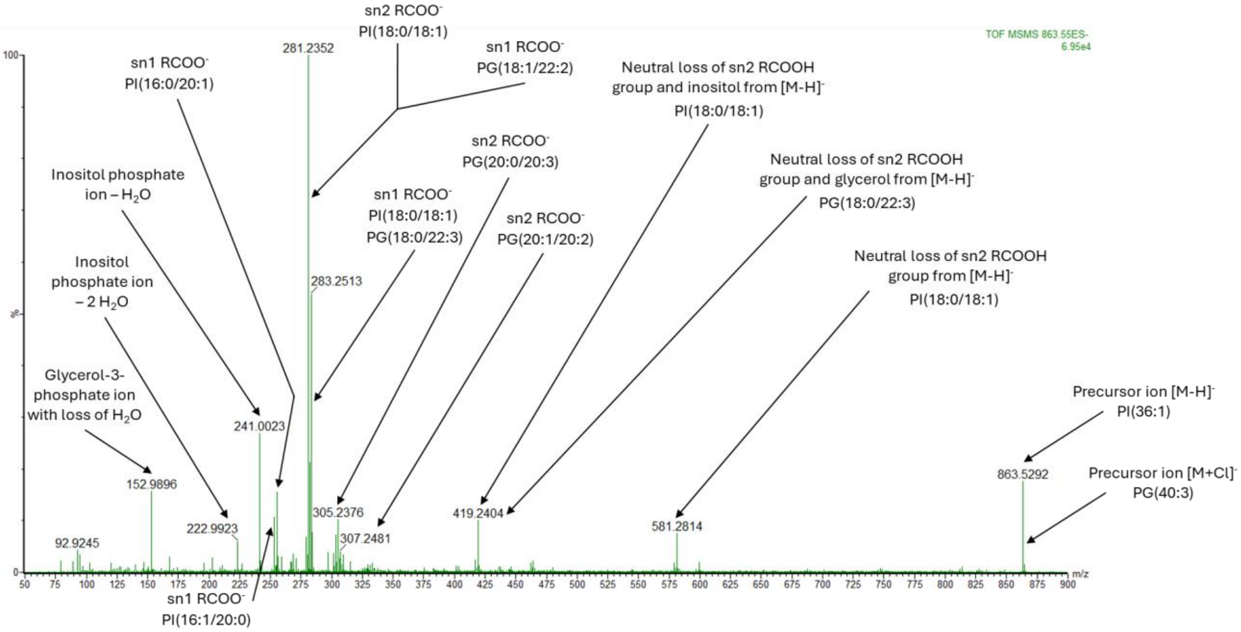

MS/MS spectrum of *m*/*z* 885.550 corresponding to PI(38:4).

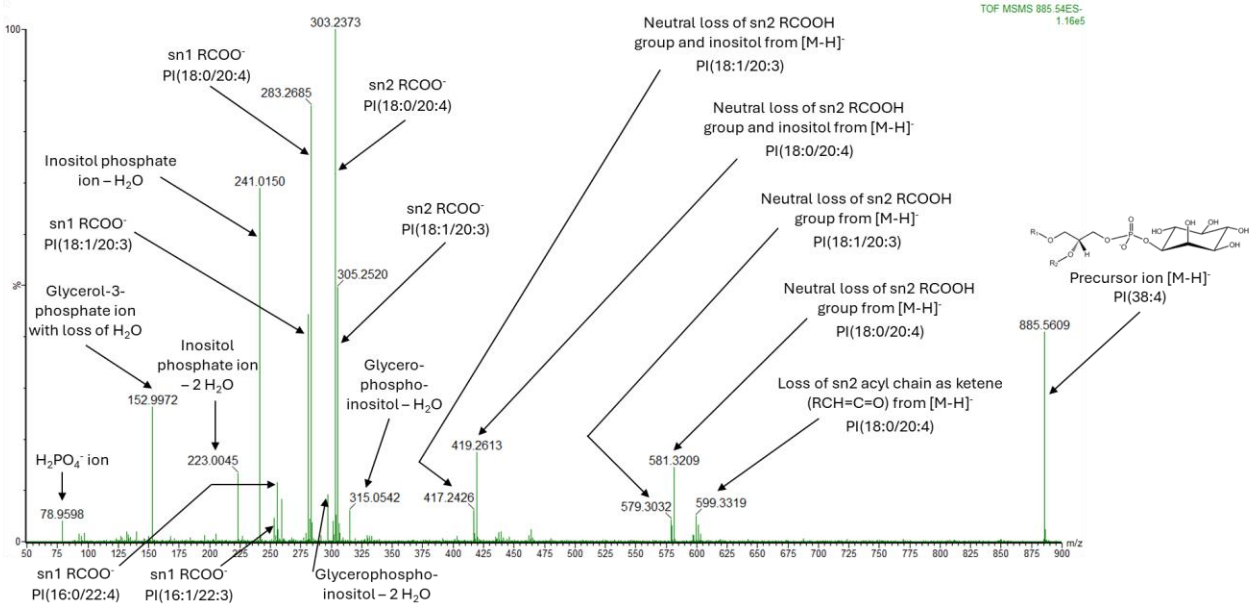

MS/MS spectrum of *m*/*z* 887.566 corresponding to PI(38:3).

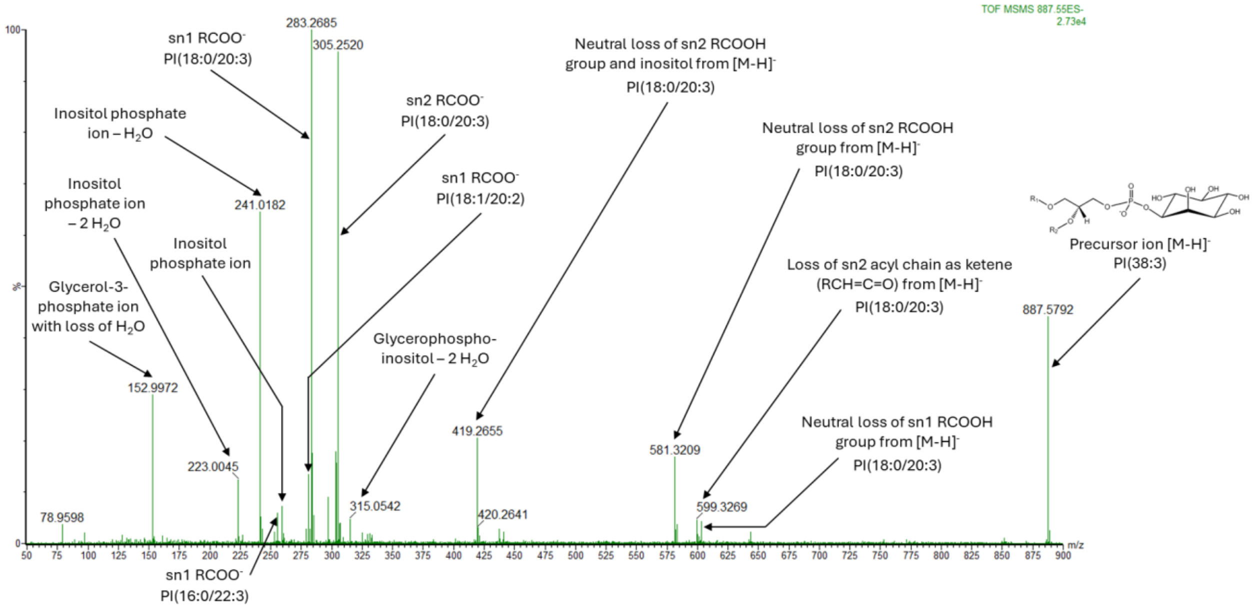

MS/MS spectrum of *m*/*z* 889.581 corresponding to PI(38:2 and PG(42:4).

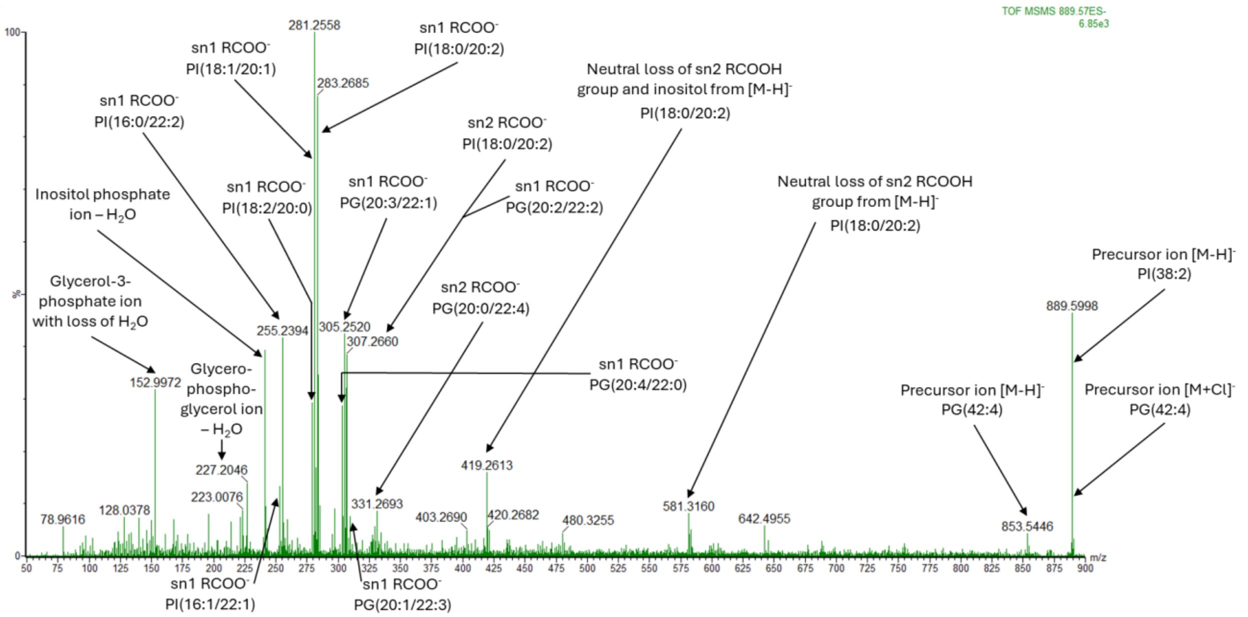

Table S1.

Major lipidomic differences identified between tumorous and non-cancerous (adjacent) parts of the breast adenocarcinoma (BA) tissue sample in the case of LA-REIMS imaging.

m/z (measured)	m/z (accurate)	Lipids	Adduct	More abundant
642.487	642.495	HexCer(30:1;O2)	$[M-H]^-$	Adjacent
678.463	678.472	HexCer(30:1;O2)	$[M+Cl]^-$	Adjacent
679.469	679.467	CerPE(32:1;O5)	$[M-H]^-$	Adjacent
680.466	680.466	CerP(38:5;O3)	$[M-H]^-$	Adjacent
687.542	687.545	CerPE(36:1;O2)	$[M-H]^-$	Adjacent
688.546	688.552	Cer(42:5;O3)	$[M-H]^-$	Adjacent
722.509	722.513	PE(O-36:5)	$[M-H]^-$	Tumorous
		PE(P-36:4)	$[M-H]^-$	Tumorous
750.543	750.544	PE(O-38:5)	$[M-H]^-$	Tumorous
		PE(P-38:4)	$[M-H]^-$	Tumorous
752.555	752.560	PE(O-38:4)	$[M-H]^-$	Tumorous
		PE(P-38:3)	$[M-H]^-$	Tumorous
766.539	766.539	PE(38:4)	$[M-H]^-$	Tumorous
885.552	885.550	PI(38:4)	$[M-H]^-$	Tumorous

Table S2.

Major lipidomic differences identified between tumorous and non-cancerous (adjacent) parts of the breast simplex tubulopapillar carcinoma (BSTC) tissue sample in the case of LA-REIMS imaging.

m/z (measured)	m/z (accurate)	Lipids	Adduct	More abundant
697.481	697.481	PA(36:3)	$[M-H]^-$	Tumorous
699.499	699.497	PA(36:2)	$[M-H]^-$	Tumorous
714.508	714.508	PE(34:2)	$[M-H]^-$	Tumorous
716.523	716.524	PE(34:1)	$[M-H]^-$	Tumorous
733.458	733.458	PA(36:3)	$[M+Cl]^-$	Tumorous
		PE(34:2)	$[M-NH_3+Cl]^-$	Tumorous
735.469	735.474	PA(36:2)	$[M+Cl]^-$	Tumorous
		PE(34:1)	$[M-NH_3+Cl]^-$	Tumorous
	735.461	PS(O-36:5)	$[M-NH_3-CH_3]^-$	Tumorous
		PS(P-36:4)	$[M-NH_3-CH_3]^-$	Tumorous
740.522	740.524	PE(36:3)	$[M-H]^-$	Tumorous
742.538	742.539	PE(36:2)	$[M-H]^-$	Tumorous
744.553	744.555	PE(36:1)	$[M-H]^-$	Tumorous

Table S3.

Major lipidomic differences identified between tumorous and non-cancerous (adjacent) parts of the skin metastasis (SM) tissue sample in the case of LA-REIMS imaging.

m/z (measured)	m/z (accurate)	Lipids	Adduct	More abundant
600.512	600.513	Cer(36:1;O2)	$[M+Cl]^{-}$	Adjacent
698.510	698.513	PE(P-34:2)	$[M-H]^{-}$	Tumorous
700.524	700.529	PE(O-34:2)	$[M-H]^{-}$	Tumorous
		PE(P-34:1)	$[M-H]^{-}$	Tumorous
714.508	714.508	PE(34:2)	$[M-H]^{-}$	Tumorous
716.523	716.524	PE(34:1)	$[M-H]^{-}$	Tumorous
722.512	722.513	PE(O-36:5)	$[M-H]^{-}$	Tumorous
		PE(P-36:4)	$[M-H]^{-}$	Tumorous
740.523	740.524	PE(36:3)	$[M-H]^{-}$	Tumorous
742.538	742.539	PE(36:2)	$[M-H]^{-}$	Tumorous
744.552	744.555	PE(36:1)	$[M-H]^{-}$	Tumorous
750.543	750.544	PE(O-38:5)	$[M-H]^{-}$	Adjacent
		PE(P-38:4)	$[M-H]^{-}$	Adjacent

Table S4.

Major lipidomic differences identified between tumorous and necrotic parts of the breast adenocarcinoma (BA) tissue sample in the case of LA-REIMS imaging.

m/z (measured)	m/z (accurate)	Lipids	Adduct	More abundant
682.590	682.591	Cer(42:2;O2)	$[M+Cl]^{-}$	Necrotic
722.509	722.513	PE(O-36:5)	$[M-H]^{-}$	Tumorous
		PE(P-36:4)	$[M-H]^{-}$	Tumorous
726.544	726.544	PE(O-36:3)	$[M-H]^{-}$	Necrotic
		PE(P-36:2)	$[M-H]^{-}$	Necrotic
748.528	748.529	PE(O-38:6)	$[M-H]^{-}$	Necrotic
		PE(P-38:5)	$[M-H]^{-}$	Necrotic
750.543	750.544	PE(O-38:5)	$[M-H]^{-}$	Tumorous
		PE(P-38:4)	$[M-H]^{-}$	Tumorous
752.555	752.560	PE(O-38:4)	$[M-H]^{-}$	Necrotic
		PE(P-38:3)	$[M-H]^{-}$	Necrotic
754.575	754.576	PE(O-38:3)	$[M-H]^{-}$	Necrotic
		PE(P-38:2)	$[M-H]^{-}$	Necrotic
766.539	766.539	PE(38:4)	$[M-H]^{-}$	Tumorous
788.554	788.553	HexCer(36:2;O6)	$[M-H]^{-}$	Necrotic
797.652	797.654	CerPE(44:2;O2)	$[M-H]^{-}$	Necrotic

Table S5.

Major lipidomic differences identified between tumorous and necrotic parts of the lung metastasis (LM) tissue sample in the case of LA-REIMS imaging.

m/z (measured)	m/z (accurate)	Lipids	Adduct	More abundant
678.465	678.472	HexCer(30:1;O2)	$[M+Cl]^-$	Necrotic
682.590	682.591	Cer(42:2;O2)	$[M+Cl]^-$	Necrotic
687.542	687.545	CerPE(36:1;O2)	$[M-H]^-$	Necrotic
742.539	742.539	PE(36:2)	$[M-H]^-$	Tumorous
748.527	748.529	PE(O-38:6)	$[M-H]^-$	Necrotic
		PE(P-38:5)	$[M-H]^-$	Necrotic
750.543	750.544	PE(O-38:5)	$[M-H]^-$	Tumorous
		PE(P-38:4)	$[M-H]^-$	Tumorous
754.576	754.576	PE(O-38:3)	$[M-H]^-$	Necrotic
		PE(P-38:2)	$[M-H]^-$	Necrotic
766.539	766.539	PE(38:4)	$[M-H]^-$	Tumorous
768.550	768.555	PE(38:3)	$[M-H]^-$	Tumorous
788.560	788.553	HexCer(36:2;O6)	$[M-H]^-$	Necrotic

Table S6.

Major lipidomic differences identified between non-cancerous (adjacent) and necrotic parts of the breast adenocarcinoma (BA) tissue sample in the case of LA-REIMS imaging.

m/z (measured)	m/z (accurate)	Lipids	Adduct	More abundant
642.486	642.495	HexCer(30:1;O2)	$[M-H]^-$	Adjacent
678.463	678.472	HexCer(30:1;O2)	$[M+Cl]^-$	Adjacent
687.544	687.545	CerPE(36:1;O2)	$[M-H]^-$	Adjacent
726.546	726.544	PE(O-36:3)	$[M-H]^-$	Necrotic
		PE(P-36:2)	$[M-H]^-$	Necrotic
728.559	728.560	PE(O-36:2)	$[M-H]^-$	Necrotic
		PE(P-36:1)	$[M-H]^-$	Necrotic
748.528	748.529	PE(O-38:6)	$[M-H]^-$	Necrotic
		PE(P-38:5)	$[M-H]^-$	Necrotic
750.542	750.544	PE(O-38:5)	$[M-H]^-$	Necrotic
		PE(P-38:4)	$[M-H]^-$	Necrotic
752.562	752.560	PE(O-38:4)	$[M-H]^-$	Necrotic
		PE(P-38:3)	$[M-H]^-$	Necrotic
754.575	754.576	PE(O-38:3)	$[M-H]^-$	Necrotic
		PE(P-38:2)	$[M-H]^-$	Necrotic
788.558	788.553	HexCer(36:2;O6)	$[M-H]^-$	Necrotic

Table S7.

Major lipidomic differences identified between tumorous and non-cancerous (adjacent) parts of the breast adenocarcinoma (BA) tissue sample in the case of DESI-MSI.

m/z (measured)	*m/z* (accurate)	Lipids	Adduct	More abundant
722.513	722.513	PE(O-36:5)<br>PE(P-36:4)	$[M-H]^-$<br>$[M-H]^-$	Tumorous<br>Tumorous
748.527	748.529	PE(O-38:6)<br>PE(P-38:5)	$[M-H]^-$<br>$[M-H]^-$	Tumorous<br>Tumorous
750.544	750.544	PE(O-38:5)<br>PE(P-38:4)	$[M-H]^-$<br>$[M-H]^-$	Tumorous<br>Tumorous
752.553	752.560	PE(O-38:4)<br>PE(P-38:3)	$[M-H]^-$<br>$[M-H]^-$	Tumorous<br>Tumorous
766.539	766.539	PE(38:4)	$[M-H]^-$	Tumorous
788.545	788.545	PS(36:1)	$[M-H]^-$	Tumorous
812.543	812.545	PS(38:3)	$[M-H]^-$	Tumorous
885.550	885.550	PI(38:4)	$[M-H]^-$	Tumorous
887.560	887.566	PI(38:3)	$[M-H]^-$	Tumorous

Table S8.

Major lipidomic differences identified between tumorous and non-cancerous (adjacent) parts of the breast simplex tubulopapillar carcinoma (BSTC) tissue sample in the case of DESI-MSI.

m/z (measured)	*m/z* (accurate)	Lipids	Adduct	More abundant
698.514	698.513	PE(P-34:2)	$[M-H]^-$	Tumorous
700.526	700.529	PE(O-34:2)<br>PE(P-34:1)	$[M-H]^-$<br>$[M-H]^-$	Tumorous<br>Tumorous
726.543	726.544	PE(O-36:3)<br>PE(P-36:2)	$[M-H]^-$<br>$[M-H]^-$	Tumorous<br>Tumorous
728.554	728.560	PE(O-36:2)<br>PE(P-36:1)	$[M-H]^-$<br>$[M-H]^-$	Tumorous<br>Tumorous
742.538	742.539	PE(36:2)	$[M-H]^-$	Tumorous
744.552	744.555	PE(36:1)	$[M-H]^-$	Tumorous
748.525	748.529	PE(O-38:6)<br>PE(P-38:5)	$[M-H]^-$<br>$[M-H]^-$	Tumorous<br>Tumorous
752.555	752.560	PE(O-38:4)<br>PE(P-38:3)	$[M-H]^-$<br>$[M-H]^-$	Tumorous<br>Tumorous
788.545	788.545	PS(36:1)	$[M-H]^-$	Tumorous
885.550	885.550	PI(38:4)	$[M-H]^-$	Tumorous

Table S9.

Major lipidomic differences identified between tumorous and non-cancerous (adjacent) parts of the skin metastasis (SM) tissue sample in the case of DESI-MSI.

m/z (measured)	*m/z* (accurate)	Lipids	Adduct	More abundant
700.527	700.529	PE(O-34:2)<br>PE(P-34:1)	$[M-H]^-$<br>$[M-H]^-$	Tumorous<br>Tumorous
716.522	716.524	PE(34:1)	$[M-H]^-$	Tumorous
740.523	740.524	PE(36:3)	$[M-H]^-$	Tumorous
742.538	742.539	PE(36:2)	$[M-H]^-$	Tumorous
744.552	744.555	PE(36:1)	$[M-H]^-$	Tumorous
750.543	750.544	PE(O-38:5)<br>PE(P-38:4)	$[M-H]^-$<br>$[M-H]^-$	Adjacent<br>Adjacent
788.545	788.545	PS(36:1)	$[M-H]^-$	Tumorous
833.518	833.519	PI(34:2)	$[M-H]^-$	Tumorous
861.550	861.550	PI(36:2)<br>PG(40:4)	$[M-H]^-$<br>$[M+Cl]^-$	Tumorous<br>Tumorous
885.550	885.550	PI(38:4)	$[M-H]^-$	Tumorous

Table S10.

Major lipidomic differences identified between non-cancerous (adjacent) and necrotic parts of the breast adenocarcinoma (BA) tissue sample in the case of DESI-MSI.

m/z (measured)	*m/z* (accurate)	Lipids	Adduct	More abundant
726.545	726.544	PE(O-36:3)<br>PE(P-36:2)	$[M-H]^-$<br>$[M-H]^-$	Necrotic<br>Necrotic
728.559	728.560	PE(O-36:2)<br>PE(P-36:1)	$[M-H]^-$<br>$[M-H]^-$	Necrotic<br>Necrotic
748.529	748.529	PE(O-38:6)<br>PE(P-38:5)	$[M-H]^-$<br>$[M-H]^-$	Necrotic<br>Necrotic
750.544	750.544	PE(O-38:5)<br>PE(P-38:4)	$[M-H]^-$<br>$[M-H]^-$	Necrotic<br>Necrotic
752.558	752.560	PE(O-38:4)<br>PE(P-38:3)	$[M-H]^-$<br>$[M-H]^-$	Necrotic<br>Necrotic
754.574	754.576	PE(O-38:3)<br>PE(P-38:2)	$[M-H]^-$<br>$[M-H]^-$	Necrotic<br>Necrotic
788.545	788.545	PS(36:1)	$[M-H]^-$	Necrotic
885.550	885.550	PI(38:4)	$[M-H]^-$	Necrotic
887.561	887.566	PI(38:3)	$[M-H]^-$	Necrotic

Table S11.

Major lipidomic differences identified between tumorous and necrotic parts of the breast adenocarcinoma (BA) tissue sample in the case of DESI-MSI.

m/z (measured)	*m/z* (accurate)	Lipids	Adduct	More abundant
726.543	726.544	PE(O-36:3)	$[M-H]^-$	Necrotic
		PE(P-36:2)	$[M-H]^-$	Necrotic
728.554	728.560	PE(O-36:2)	$[M-H]^-$	Necrotic
		PE(P-36:1)	$[M-H]^-$	Necrotic
744.552	744.555	PE(36:1)	$[M-H]^-$	Necrotic
748.525	748.529	PE(O-38:6)	$[M-H]^-$	Necrotic
		PE(P-38:5)	$[M-H]^-$	Necrotic
750.544	750.544	PE(O-38:5)	$[M-H]^-$	Tumorous
		PE(P-38:4)	$[M-H]^-$	Tumorous
752.555	752.560	PE(O-38:4)	$[M-H]^-$	Necrotic
		PE(P-38:3)	$[M-H]^-$	Necrotic
754.574	754.576	PE(O-38:3)	$[M-H]^-$	Necrotic
		PE(P-38:2)	$[M-H]^-$	Necrotic
766.540	766.539	PE(38:4)	$[M-H]^-$	Tumorous
788.545	788.545	PS(36:1)	$[M-H]^-$	Necrotic
885.550	885.550	PI(38:4)	$[M-H]^-$	Tumorous
889.580	889.581	PI(38:2)	$[M-H]^-$	Necrotic
		PG(42:2)	$[M-H]^-$	Necrotic

Table S12.

Major lipidomic differences identified between tumorous and necrotic parts of the lung metastasis (LM) tissue sample in the case of DESI-MSI.

m/z (measured)	*m/z* (accurate)	Lipids	Adduct	More abundant
726.543	726.544	PE(O-36:3)	$[M-H]^-$	Necrotic
		PE(P-36:2)	$[M-H]^-$	Necrotic
728.554	728.560	PE(O-36:2)	$[M-H]^-$	Necrotic
		PE(P-36:1)	$[M-H]^-$	Necrotic
744.552	744.555	PE(36:1)	$[M-H]^-$	Necrotic
748.525	748.529	PE(O-38:6)	$[M-H]^-$	Necrotic
		PE(P-38:5)	$[M-H]^-$	Necrotic
750.544	750.544	PE(O-38:5)	$[M-H]^-$	Tumorous
		PE(P-38:4)	$[M-H]^-$	Tumorous
754.574	754.576	PE(O-38:3)	$[M-H]^-$	Necrotic
		PE(P-38:2)	$[M-H]^-$	Necrotic
766.540	766.539	PE(38:4)	$[M-H]^-$	Tumorous
788.545	788.545	PS(36:1)	$[M-H]^-$	Necrotic
885.550	885.550	PI(38:4)	$[M-H]^-$	Tumorous
889.580	889.581	PI(38:2)	$[M-H]^-$	Necrotic
		PG(42:2)	$[M-H]^-$	Necrotic

Table S13.

The most abundant lipids identified for the breast adenocarcinoma (BA) and its lung metastasis (LM) cell lines in the case of LA-REIMS imaging.

m/z (measured)	*m/z* (accurate)	Lipids Lara	Adduct
671.464	671.466	PA(34:2)	$[M-H]^-$
673.480	673.481	PA(34:1)	$[M-H]^-$
699.496	699.497	PA(36:2)	$[M-H]^-$
701.514	701.513	PA(36:1)	$[M-H]^-$
716.522	716.524	PE(34:1)	$[M-H]^-$
725.513	725.513	PA(38:3)	$[M-H]^-$
727.529	727.528	PA(38:2)	$[M-H]^-$
735.472	735.474	PA(36:2)<br>PE(34:1)	$[M+Cl]^-$<br>$[M-NH_3+Cl]^-$
737.479	737.489	PA(36:1)	$[M+Cl]^-$
742.537	742.539	PE(36:2)	$[M-H]^-$
744.552	744.555	PE(36:1)	$[M-H]^-$
748.524	748.529	PE(O-38:6)<br>PE(P-38:5)	$[M-H]^-$<br>$[M-H]^-$
750.539	750.544	PE(O-38:5)<br>PE(P-38:4)	$[M-H]^-$<br>$[M-H]^-$
761.488	761.489	PA(38:3)<br>PS(O-38:6)<br>PS(P-38:5)	$[M-H]^-$<br>$[M-NH_3-CH_3]^-$<br>$[M-NH_3-CH_3]^-$
766.532	766.539	PE(38:4)	$[M-H]^-$

Table S14.

The most abundant lipids identified for the breast simplex tubulopapillar carcinoma (BSTC) and its skin metastasis (SM) cell lines in the case of LA-REIMS imaging.

m/z (measured)	*m/z* (accurate)	Lipids NM	Adduct
673.480	673.481	PA(34:1)	$[M-H]^-$
699.496	699.497	PA(36:2)	$[M-H]^-$
701.510	701.513	PA(36:1)	$[M-H]^-$
723.497	723.497	PA(38:4)	$[M-H]^-$
725.510	725.513	PA(38:3)	$[M-H]^-$
727.526	727.528	PA(38:2)	$[M-H]^-$
735.477	735.474	PA(36:2)<br>PE(34:1)	$[M+Cl]^-$<br>$[M-NH_3+Cl]^-$
744.552	744.555	PE(36:1)	$[M-H]^-$
747.503	747.497	PA(40:6)<br>PE(38:5)	$[M-H]^-$<br>$[M-NH_4]^-$
748.520	748.529	PE(O-38:6)<br>PE(P-38:5)	$[M-H]^-$<br>$[M-H]^-$
749.514	749.513	PA(40:5)<br>PE(38:4)	$[M-H]^-$<br>$[M-NH_4]^-$
750.533	750.544	PE(O-38:5)<br>PE(P-38:4)	$[M-H]^-$<br>$[M-H]^-$
751.529	751.528	PA(40:4)<br>PE(38:3)	$[M-H]^-$<br>$[M-NH_4]^-$
766.536	766.539	PE(38:4)	$[M-H]^-$
885.549	885.550	PI(38:4)	$[M-H]^-$

Table S15.

The most abundant lipids identified for the breast adenocarcinoma (BA) and its lung metastasis (LM) cell lines in the case of DESI-MSI.

m/z (measured)	*m/z* (accurate)	Lipids Lara	Adduct
701.517	701.513	PA(36:1)	$[M-H]^-$
727.533	727.528	PA(38:2)	$[M-H]^-$
742.538	742.539	PE(36:2)	$[M-H]^-$
744.552	744.555	PE(36:1)	$[M-H]^-$
747.516	747.518	PG(34:1)	$[M-H]^-$
748.525	748.529	PE(O-38:6)<br>PE(P-38:5)	$[M-H]^-$<br>$[M-H]^-$
750.541	750.544	PE(O-38:5)<br>PE(P-38:4)	$[M-H]^-$<br>$[M-H]^-$
774.538	774.544	PE(P-40:6)	$[M-H]^-$
775.542	775.550	PG(36:1)	$[M-H]^-$
788.543	788.545	PS(36:1)	$[M-H]^-$
819.517	819.518	PG(40:7)	$[M-H]^-$
861.548	861.550	PI(36:2)<br>PG(40:4)	$[M-H]^-$<br>$[M+Cl]^-$
885.549	885.550	PI(38:4)	$[M-H]^-$
887.564	887.566	PI(38:3)	$[M-H]^-$
889.575	889.581	PI(38:2)<br>PG(42:2)	$[M-H]^-$<br>$[M+Cl]^-$

Table S16.

The most abundant lipids identified for the breast simplex tubulopapillar carcinoma (BSTC) and its skin metastasis (SM) cell lines in the case of DESI-MSI.

m/z (measured)	*m/z* (accurate)	Lipids	Adduct
744.551	744.555	PE(36:1)	$[M-H]^-$
748.524	748.529	PE(O-38:6)<br>PE(P-38:5)	$[M-H]^-$<br>$[M-H]^-$
749.522	749.513	PA(40:5)	$[M-H]^-$
750.537	750.544	PE(O-38:5)<br>PE(P-38:4)	$[M-H]^-$<br>$[M-H]^-$
751.536	751.528	PA(40:4)	$[M-H]^-$
766.537	766.539	PE(38:4)	$[M-H]^-$
773.530	773.534	PG(36:2)	$[M-H]^-$
774.536	774.544	PE(P-40:6)	$[M-H]^-$
775.542	775.541	PG(36:1)	$[M-H]^-$
776.550	776.560	PE(P-40:5)	$[M-H]^-$
788.540	788.545	PS(36:1)	$[M-H]^-$
812.535	812.545	PS(38:3)	$[M-H]^-$
819.516	819.518	PG(40:7)	$[M-H]^-$
885.547	885.550	PI(38:4)	$[M-H]^-$
887.559	887.566	PI(38:3)	$[M-H]^-$
